# Changes in Ect2 Localization Couple Actomyosin-Dependent Cell Shape Changes to Mitotic Progression

**DOI:** 10.1016/j.devcel.2012.06.003

**Published:** 2012-08-14

**Authors:** Helen K. Matthews, Ulysse Delabre, Jennifer L. Rohn, Jochen Guck, Patricia Kunda, Buzz Baum

**Affiliations:** 1 MRC Laboratory for Molecular Cell Biology, University College London, Gower St., London WC1E 6BT, UK; 2 Department of Physics, Cavendish Laboratory, University of Cambridge, J.J. Thomson Avenue, Cambridge CB3 0HE, UK; 3 PCC Curie, Institut Curie/CNRS/Université Paris 6 - UMR 168, 26 rue d'Ulm, 75248 Paris, France

## Abstract

As they enter mitosis, animal cells undergo profound actin-dependent changes in shape to become round. Here we identify the Cdk1 substrate, Ect2, as a central regulator of mitotic rounding, thus uncovering a link between the cell-cycle machinery that drives mitotic entry and its accompanying actin remodeling. Ect2 is a RhoGEF that plays a well-established role in formation of the actomyosin contractile ring at mitotic exit, through the local activation of RhoA. We find that Ect2 first becomes active in prophase, when it is exported from the nucleus into the cytoplasm, activating RhoA to induce the formation of a mechanically stiff and rounded metaphase cortex. Then, at anaphase, binding to RacGAP1 at the spindle midzone repositions Ect2 to induce local actomyosin ring formation. Ect2 localization therefore defines the stage-specific changes in actin cortex organization critical for accurate cell division.

## Highlights

► Ect2 drives dynamic changes in cell shape throughout mitosis ► Ect2 induces actin-dependent changes in cortical mechanics at mitotic onset ► Ect2's distinct functions are achieved through changes in subcellular localization ► Actin remodeling for animal cell division begins at mitotic entry

## Teaser

Cell division is accompanied by dramatic changes in cell shape, which Matthews et al. show are regulated by the Cdk1 substrate Ect2. At mitotic onset, Ect2 leaves the nucleus to activate RhoA and cortical actomyosin, driving mitotic rounding. It is then repositioned at mitotic exit to drive actomyosin ring formation.

## Introduction

Cell division requires sequential changes in cell architecture, which are coordinated by a small set of conserved mitotic kinases (Ma and Poon, 2011). Although most recent attention has focused on the changes in microtubule organization that accompany spindle assembly and that drive chromosome segregation, mitotic progression is also accompanied by profound changes in cell shape. These begin at the onset of mitosis as cells detach from the substrate and round up (Cramer and Mitchison, 1997; Harris, 1973); a process that is important for spindle assembly, positioning, and chromosome capture (Carreno et al., 2008; Kunda and Baum, 2009; Kunda et al., 2008). At mitotic exit, cells then elongate and divide in two, before respreading to take up their resting interphase shape once more.

Mitotic rounding requires the loss of substrate adhesion (Dao et al., 2009), together with changes in surface volume ratio and osmotic pressure (Stewart et al., 2011). In addition, the actin cytoskeleton is completely remodeled to generate a rigid and rounded actomyosin cortex (Kunda et al., 2008). Although several actin regulators, including ERM proteins (Carreno et al., 2008; Kunda et al., 2008), myosin II (Maddox and Burridge, 2003), Cofilin, and WDR1 (Fujibuchi et al., 2005), are known to play roles in this process, it is not known how changes in actin organization are coupled to mitotic entry and progression.

Entry into mitosis in mammalian cells is controlled by activation of the mitotic Cdk1/CyclinB complex, through a tightly regulated process that involves multiple feedback loops (Lindqvist et al., 2009). Once active, Cdk1/CyclinB phosphorylates a large number of targets, triggering many of the early events of mitosis including centrosome separation, chromosome condensation, and nuclear envelope breakdown (Gavet and Pines, 2010b). Although some of the key substrates mediating the effects of Cdk1/CyclinB activity on chromatin, the nuclear lamina, and the mitotic spindle have been identified (Blangy et al., 1995; Kimura et al., 1998; Peter et al., 1990), it is not known if changes in Cdk1/CyclinB activity also function to directly alter actin filament organization and dynamics.

Here, we identify a known Cdk1 substrate (Hara et al., 2006; Niiya et al., 2006), Ect2, as a regulator of mitotic rounding. Ect2 is a RhoGEF that was previously shown to be essential for cytokinesis (Tatsumoto et al., 1999), where it activates RhoA to regulate assembly of the actomyosin contractile ring (Chalamalasetty et al., 2006; Nishimura and Yonemura, 2006; Yüce et al., 2005). We show that Ect2 also activates RhoA and its downstream effectors, Rho kinase (ROK), and myosin II, at the onset of mitosis to induce the actomyosin remodeling that drives both mitotic rounding and cortical stiffening. This early function of Ect2 is dependent on its export from the nucleus in prophase, but does not require RacGAP1 (also known as MgcRacGAP) or microtubules, which have been shown to drive the relocalization of Ect2 to the cell equator prior to cytokinesis (Burkard et al., 2009; Petronczki et al., 2007; Somers and Saint, 2003; Wolfe et al., 2009; Yüce et al., 2005). These data show that, through regulated changes in its localization, Ect2 is able to reshape the mitotic cell to drive rounding upon entry into mitosis and cleavage furrow formation at mitotic exit.

## Results

### Rounding Is Initiated at the Start of Mitosis

**Figure 1:**
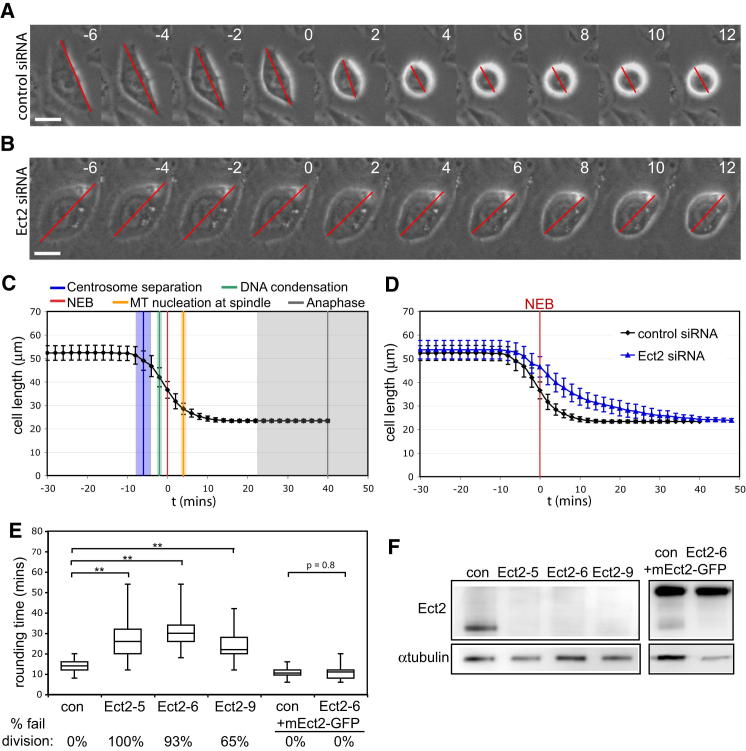
Ect2 Alters the Dynamics of Mitotic Cell Rounding (A and B) Time-lapse phase contrast images of HeLa cells rounding up before mitosis with cell length (Feret's diameter) indicated by red line showing cells treated with (A) control siRNA and (B) Ect2 siRNA. Images taken every 2 min. Scale bar, 20 μm. (C) Mean length of 22 cells during progression through mitosis, aligned so that time point 0 represents nuclear envelope breakdown (NEB). Error bars denote SD. Colored vertical lines show mean timing of mitotic events with shaded areas showing SD. Mitotic events were visualized using the expression of histone H2B-mRFP (for chromatin condensation and anaphase) and tubulin-GFP (centrosome separation and microtubule nucleation at spindle). NEB was recorded as the time point at which free tubulin-GFP dimers are able to enter the nucleus. (D) Comparison of rounding in cells treated with control siRNA (n = 20 cells) and Ect2 siRNA (n = 23). Error bars denote SD. (E) Box plot showing time taken to round up at mitosis for control siRNA (n = 31) compared to three nonoverlapping siRNAs targeting Ect2 (n = 27, 33, and 25) in control HeLa cells, and in HeLa cells expressing mouse Ect2-GFP at endogenous levels (n = 28 cells in each condition). Central line shows median, boxes are quartiles and whiskers show complete range. Cells were imaged 24 hr post-RNAi and only the first division after RNAi treatment was analyzed. The percentage of cells that then go on to fail cytokinesis in each condition is indicated. (F) Western blot showing knockdown of human Ect2 but not mouse Ect2-GFP (upper band) by three siRNAs targeting Ect2. See also Figure S1.

To better understand the coupling between cell shape changes and mitotic progression, we began by determining the timing of mitotic rounding relative to the other events of mitotic entry. HeLa cells expressing Histone H2B-mRFP and tubulin-GFP (Steigemann et al., 2009) were imaged every 2 min as they progressed through the cell cycle. Cell length (Feret's diameter) was measured (Figure 1A) and used to compare the timing of mitotic rounding with that of centrosome separation, chromosome condensation, nuclear envelope breakdown, and spindle assembly (Figure 1C). Mitotic rounding lasted 13.6 ± 1.8 min, during which time mean HeLa cell length (Picone et al., 2010) was reduced from 53 ± 3 μm to 23.5 ± 1.3 μm. Rounding began in early prophase, before centrosome separation and visible chromatin condensation, around 6 min before nuclear envelope breakdown (Figure 1C). This makes rounding one of the earliest events in mitosis, concordant with the rise in Cdk1 activity during prophase as measured using a FRET probe (Gavet and Pines, 2010b). Since Cdk1/CyclinB is thought to directly control many events of prophase (Gavet and Pines, 2010a), this suggested the possibility that Cdk1/Cyclin B also instigates mitotic rounding. Indeed, Cdk1/Cyclin B has been shown to be sufficient to induce rounding upon injection into interphase cells (Lamb et al., 1990). Thus, in our search for upstream regulators of cell rounding, we focused our attention on established Cdk1 substrates.

### Ect2 Controls the Dynamics of Mitotic Rounding

To identify regulators of mitotic rounding, we carried out an RNAi screen. We used siRNA to silence 60 key actin regulators. Since we aimed to identify genes that couple mitotic progression to changes in cell shape, this set specifically included siRNAs targeting actin regulators previously identified as mitotic kinase substrates in systematic large-scale screens (Beausoleil et al., 2006; Blethrow et al., 2008; Dephoure et al., 2008; Ji et al., 2002). Two days after siRNA treatment, HeLa cells were fixed, stained, and analyzed to identify gene-specific siRNAs that induced reproducible changes in mitotic cell shape and actin organization (for details of screen methodology and list of genes screened, see Supplemental Experimental Procedures and Table S1 available online). This identified a number of siRNAs that affected mitotic cell shape. Unsurprisingly in light of previous work (Fujibuchi et al., 2005), this included two inhibitors of actin filament formation, WDR1 and actin capping protein (Table S1). More significantly for our purposes, the screen also identified a single well-established Cdk1 substrate, Ect2.

To confirm a role for Ect2 in mitotic rounding we turned to time-lapse microscopy. Ect2 loss of function has previously been shown cause cytokinesis failure, leading to the formation of binucleate cells (Tatsumoto et al., 1999). Our analysis therefore focused on the first cell division after Ect2 knockdown (around 24 hr after RNAi treatment) to avoid delays in rounding caused by cells being large and multinucleate. This analysis revealed that Ect2 depleted cells initiate rounding on schedule relative to nuclear envelope breakdown, but round more slowly (mean rounding time of 31.1 ± 4.3 min, Figures 1B and 1D). The vast majority then went on to fail cytokinesis (Figure 1E). Both phenotypes were replicated using three different nonoverlapping siRNAs (Figure 1E) and the knockdown at 24 hr was verified in each case by western blotting (Figure 1F). Furthermore, as a definitive proof that the phenotype reflects depletion of Ect2 itself, we rescued the RNAi phenotype in HeLa cells using the constitutive expression of mouse Ect2-GFP (Hutchins et al., 2010), which lacks the siRNA binding sequence (Figures 1E and 1F). Finally, this function for Ect2 is not confined to HeLa cells, as a similar phenotype was observed following Ect2 knockdown in RPE1 cells, a diploid nontransformed human cell line, as well as in *Drosophila* S2R+ cells depleted of the fly Ect2 homolog, *pebble* (Prokopenko et al., 1999) (Figure S1). These data lead us to conclude that Ect2 plays a conserved role in mitotic rounding.

### Ect2 Is Required for Assembly of a Stiff Cortical Actin Cytoskeleton at Mitosis

**Figure 2:**
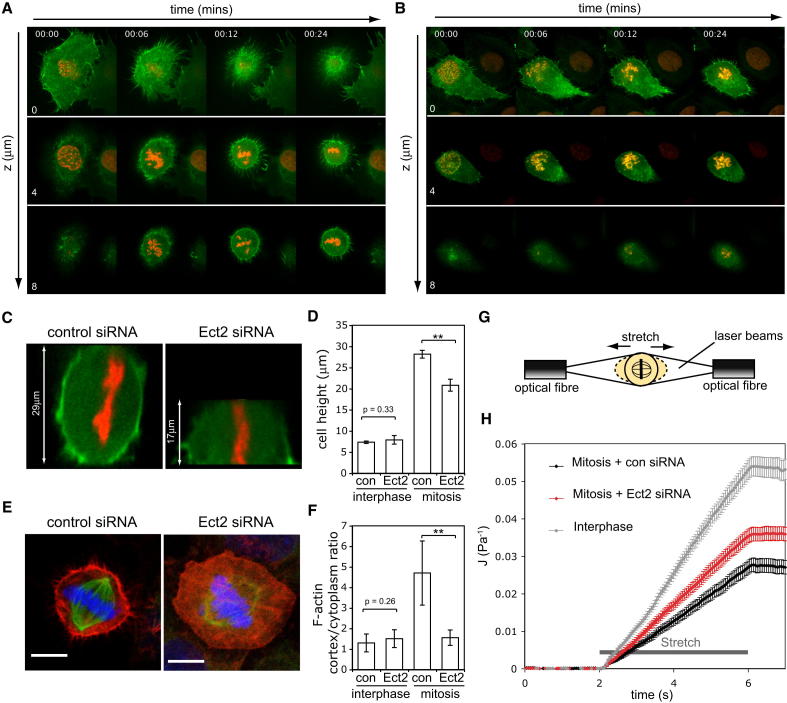
Ect2 Is Required for the Organization of a Rigid, Cortical Actin Cytoskeleton in Mitosis (A and B) Time-lapse confocal images of HeLa cells entering mitosis labeled with LifeAct-GFP and histone H2B-mRFP treated with control siRNA (A) and Ect2 siRNA (B). Time is in minutes. Three different z planes, 4 μm apart are shown. See also Movie S1. (C) XZ projections of metaphase cells labeled with LifeAct-GFP and histone H2B-mRFP. Confocal Z sections were taken every 200 nm through living cells covering the full height of the cell. (D) Graph showing the mean height of cells in interphase and metaphase treated with control siRNA or Ect2 siRNA (n = 10–15 cells per condition). Error bars show SD. (E) Confocal micrographs of fixed metaphase HeLa cells stained to show the actin cytoskeleton in control siRNA and Ect2 siRNA cells. Actin is labeled with phalloidin-TRITC in red, tubulin in green and 4′,6-diamidino-2-phenylindole (DAPI) in blue, scale bars 10 μm. (F) Quantification of the ratio of cortical/cytoplasmic actin in control and Ect2 RNAi cells in interphase and mitosis. Mean signal intensity in a 3 × 3 pixel box was measured in the actin channel at two locations: 0.5 μm from the cell edge (cortex) and 5 μm from the cell edge (cytoplasm). Four sites per cell were measured and the graph shows the mean values for 15 cells per condition with error bars denoting SD. (G) Diagram of the optical stretcher set-up used to measure cell compliance. (H) Graph showing mean compliance J(t) (see Experimental Procedures for detail) over time as cells are subjected to optical stretching for 4 s comparing control siRNA cells in interphase (n = 60 cells) and mitosis (n = 63) and Ect2 siRNA mitotic cells (n = 45). Error bars denote SEM. See also Figure S2.

Since the actin cytoskeleton controls mitotic cell shape (Kunda and Baum, 2009), we used confocal time-lapse microscopy to determine whether this role for Ect2 in rounding reflects a role in mitotic actin remodeling. In control cells expressing LifeAct-GFP (Riedl et al., 2008), actin filaments were found to redistribute to form a visible cortex underlying the plasma membrane as cells rounded and increased in height upon entry into mitosis (Figure 2A; Movie S1). This cortical recruitment was clearly perturbed in both live (Figure 2B; Movie S1) and fixed metaphase Ect2 RNAi cells (Figures 2E and 2F). First actin filaments appeared profoundly disorganized in Ect2 RNAi cells (Figure 2E). In addition, Ect2 RNAi cells were considerably flatter than metaphase control cells (Figures 2C and 2D).

This role for Ect2 in mitotic actin cortex assembly would be expected to lead to corresponding changes in mitotic cell mechanics (Kunda et al., 2008). To test whether or not this is the case, we used an “optical stretcher” to measure the rigidity of control and Ect2 RNAi cells in mitosis. This phototonic tool consists of two counter-propagating laser beams that are used to trap and exert a stretching force on suspended cells passing through a central microfluidic chamber (Figure 2G) (Guck et al., 2001). The deformation induced by the two beams can then be used to determine a cell's compliance, an inverse measure of its stiffness. Using this system, we first established that, as previously reported (Kunda et al., 2008), mitotic cells are less compliant than interphase cells and that this depends on an intact actin cytoskeleton (Figure S2). Strikingly, however, mitotic Ect2 RNAi cells were significantly more compliant than control cells in mitosis (Figure 2H). Taken together, these data show that Ect2 is essential for the assembly of the normal, rigid, actomyosin-based mitotic cortex.

It has previously been shown that a rigid and rounded actin cortex is essential for spindle assembly in *Drosophila* cells (Carreno et al., 2008; Kunda et al., 2008). In addition, myosin II has been shown to play an important role in centrosome separation (Rosenblatt et al., 2004). Therefore Ect2 depleted HeLa cells might be expected to exhibit spindle defects. We found that while Ect2 RNAi cells were ultimately able to build a bipolar spindle, cells suffered delays in centrosome separation and spindle assembly (Figure S2) similar to those previously observed when myosin activity is compromised (Rosenblatt et al., 2004). In addition, Ect2 RNAi cells exhibited defects in the alignment of chromosomes at the metaphase plate and in their segregation at anaphase, where we frequently observed lagging chromosomes (Figure S2). Furthermore, when we used RNAi mediated depletion of Mad2 to compromise the spindle checkpoint and to accelerate mitotic progression (Jones et al., 2004), the majority of Ect2 depleted cells exhibited catastrophic defects in chromosome segregation (Figure S2), while few defects were seen in Mad2 RNAi control cells. Thus, the Ect2 dependent changes in mitotic actin cytoskeletal organization and cell shape are required to support the timely assembly of a functional bipolar spindle.

### Ect2 Acts Upstream of RhoA and Myosin II to Drive Mitotic Rounding

**Figure 3:**
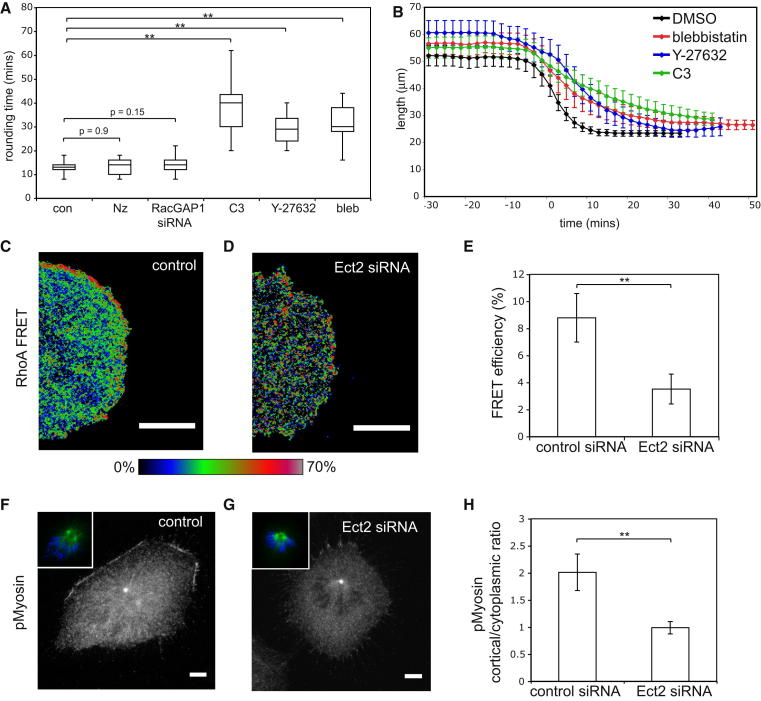
Ect2 Controls Mitotic Rounding via RhoA, Rho Kinase, and Myosin II (A) Box plot comparing rounding times of control cells (n = 22 cells) compared to cells treated with 100 ng/ml nocodozole (Nz) to depolymerize microtubules (n = 16), RacGAP1 siRNA (n = 20), 2 μg/ml C3 transferase to inhibit Rho (n = 22), 50 μM ROK inhibitor Y-27632 (n = 18) and 50 μM blebbistatin to inhibit myosin II (n = 25). Central line shows median, boxes are quartiles, and whiskers show complete range. (B) Graph showing the cell length through time for conditions in Figure 3A. Error bars denote SD. (C and D) Pseudo-colored FRET ratio images showing RhoA activity in cells arrested in prometaphase by treatment with 5 μM STLC, comparing a control siRNA cell (C) to an Ect2 siRNA treated cell (D). (E) Graph showing mean total RhoA FRET efficiency in control siRNA cells (n = 20) and Ect2 siRNA (n = 16) cells. FRET efficiency was calculated using acceptor photo-bleaching (see Experimental Procedures). Error bars denote SD. (F and G) Representative confocal images of control (F) and Ect2 siRNA (G) prometaphase cells during mitotic rounding stained for phospho-myosin light chain. Insets show tubulin staining and DNA (DAPI stain, blue). (H) Quantification of the Ect2 siRNA p-myosin II phenotype. The ratio of cortical/cytoplasmic phospho-myosin was calculated by measuring mean signal intensity in a 3 × 3 pixel box at four locations at the cortex of the cell, and four locations 5 μm into the cytoplasm. The graph shows the mean values for 11 cells per condition with error bars denoting SD. Scale bars, 5 μm. See also Figure S3.

Ect2 is essential for cytokinesis. It is recruited to the spindle midzone at anaphase through a physical interaction with a component of the centralspindlin complex, RacGAP1 (Somers and Saint, 2003; Yüce et al., 2005), where it induces the local activation of RhoA and actomyosin ring formation (Tatsumoto et al., 1999; Kamijo et al., 2006; Nishimura and Yonemura, 2006). We used small molecule inhibitors and siRNAs to determine which, if any, of these factors function together with Ect2 in mitotic rounding (Figure 3A). Neither treatment with an siRNA against RacGAP1 nor the removal of microtubules with nocodozole affected the rate of rounding. This was the case even though RacGAP1 silencing resulted in a highly penetrant failure in cytokinesis. In contrast, and as expected based on previous work, the inhibition of downstream targets of Ect2, Rho (Maddox and Burridge, 2003), ROK (Meyer et al., 2011), and myosin II (Cramer and Mitchison, 1997) led to a profound delay in mitotic rounding, similar to that seen following Ect2 RNAi (Figure 3B).

To test whether Ect2 is directly responsible for RhoA activation before the onset of anaphase, a RhoA FRET probe was used (Pertz et al., 2006). While RhoA activity was seen at the cortex of control cells in prometaphase (Figure 3C) as previously described (Mali et al., 2010; Yoshizaki et al., 2003), Ect2 silencing resulted in a marked reduction in cortical RhoA activity (Figures 3D and 3E). Since RhoA activates ROK to alter myosin II activity, in part through the phosphorylation of myosin light chain (Amano et al., 1996), we then used an antibody raised against p-myosin II to determine whether Ect2 also influences myosin II activation at the onset of mitosis. In interphase cells, p-myosin II was visible in stress fibers, which were lost along with focal adhesions in early prophase in control and Ect2 RNAi cells (Figure S3). At the same time, p-myosin II was seen accumulating at the retracting margins of control cells as they rounded up (Figure 3F), but was largely absent from Ect2 RNAi cells (Figures 3G and 3H). By contrast, when we examined ERM protein activation using the same approach (Kunda et al., 2008), we observed no differences in ERM phosphorylation between control and Ect2 RNAi cells (Figure S3). Taken together, these data reveal that Ect2 is required at the early stages of mitosis to activate RhoA and Myosin II to drive the actomyosin contraction required for cell rounding. Significantly, however, the upstream regulators of Ect2 activity are distinct from those that are required for contractile ring formation at mitotic exit.

### Ect2 Is Phosphorylated throughout Mitosis

**Figure 4:**
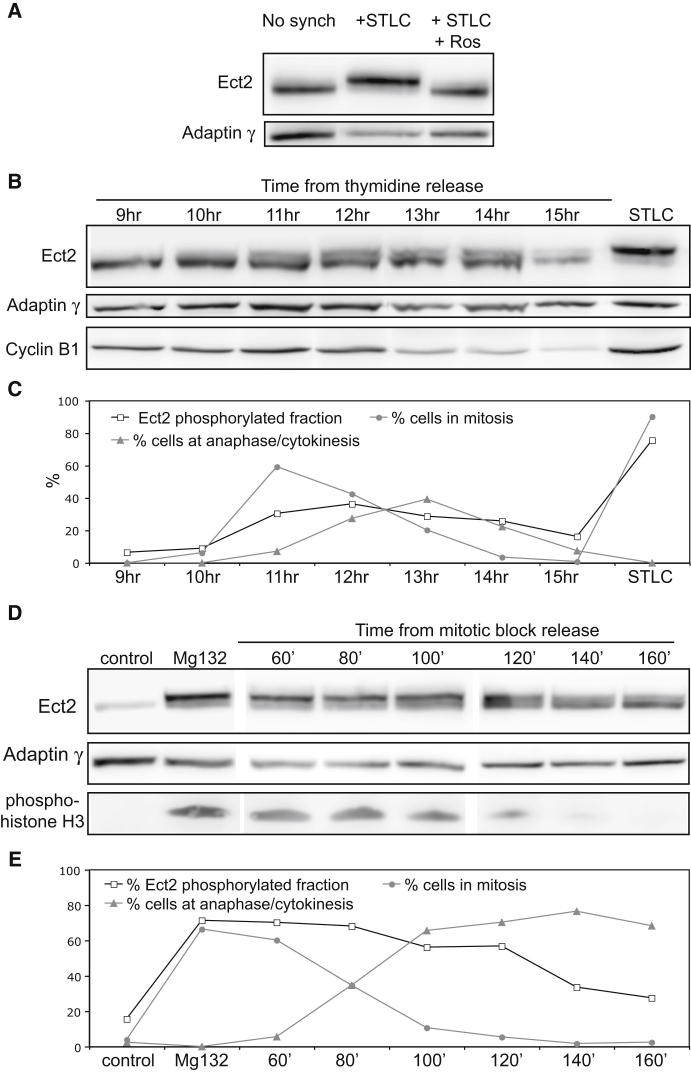
Ect2 Is Phosphorylated throughout Mitosis (A) Gel showing the band shift of Ect2 at mitosis comparing unsynchronized cells (first lane) to cells synchronized at prometaphase by an 18 hr treatment with 5 μM S-trityl-L-cysteine (STLC, second lane). This shift is abolished by addition of 50 μM Roscovitine for 2 hr (third lane), which also reverses cell rounding. (B) Band shift of Ect2 protein over a time course from 9 to 15 hr after release from double thymidine block. Image shown is representative of all experiments (n = 3). (C) Quantification of the gel in (B), showing the percentage of Ect2 protein that is phosphorylated and the percentage of cells in mitosis at each time point. The fraction of phosphorylated Ect2 was calculated by normalizing to background and then dividing the band volume for the phospho-species by the total Ect2 band volume. Mitotic stages were determined by visual inspection of the spindle and DNA following fixation and immunostaining with tubulin and DAPI of a sample of cells at each time point (n = 79–221 cells for each time point) “% cells in mitosis” includes cells in prophase, prometaphase, and metaphase. (D) Phospho-band shift of Ect2 in a synchronized population of cells as they exit mitosis after release from a metaphase arrest. This experiment was repeated twice and the image is representative of both experiments. (E) Quantification of the gel in D, showing the percentage of Ect2 phosphorylated protein compared to the percentage of cells in mitosis (prophase, prometaphase, and metaphase) and at anaphase/cytokinesis.

How is Ect2 able to control distinct processes at different times in mitosis? Ect2 has previously been shown to be phosphorylated at multiple sites during mitosis, including at several Cdk1 target sites (Hara et al., 2006; Niiya et al., 2006; Su et al., 2011; Yüce et al., 2005). This phosphorylation is required for its GEF activity (Tatsumoto et al., 1999), and has been proposed to regulate changes in Ect2 activity. This led us to examine the phosphorylation status of Ect2 during mitotic progression. Phosphorylated mitotic Ect2 migrates on a gel as a high molecular weight band (Tatsumoto et al., 1999) that is rapidly abolished following the inhibition of Cdk1 activity by Roscovitine treatment (Figure 4A). Using this gel mobility shift assay, we analyzed the extent of Ect2 phosphorylation in a synchronized population of cells following their release from a double thymidine block (Figure 4B). Phosphorylated Ect2 first appeared as synchronized cells entered mitosis 11 hr after block release, consistent with this form of the protein having an active role in mitotic rounding. Importantly, Ect2 then remained phosphorylated until 14 hr post-release, by which time the majority of cells had exited mitosis (Figure 4C). A similar time course was observed following release from a metaphase block imposed using nocodozole followed by MG132 (Figures 4D and 4E). Again, the phospho-shifted form of Ect2 visible in metaphase remained until almost all cells had completed anaphase (Figure 4E). These data show that Ect2 remains phosphorylated throughout mitosis.

### Ect2 Leaves the Nucleus in Early Mitosis

**Figure 5:**
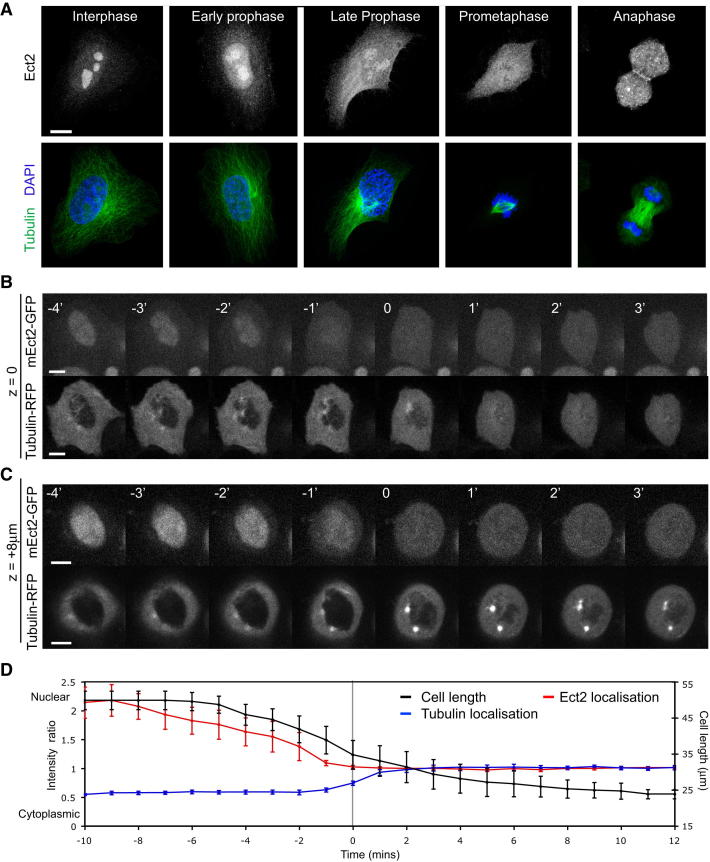
Ect2 Is Exported from the Nucleus in Early Mitosis (A) Confocal micrograph showing Ect2 localization at each stage of mitosis in fixed cells stained with an antibody against Ect2 (upper panel) and tubulin and DAPI to show mitotic stage (lower panel). Scale bar applies to all images, 10 μm. (B and C) Time-lapse confocal images of a HeLa cell entering mitosis expressing mouse Ect2-GFP (upper panels) and tubulin-RFP (lower panels). Mouse Ect2 is constitutively expressed in a BAC under its endogenous promotor (Hutchins et al., 2010). Two different z planes are shown: the bottom of the cell to show the full extent of the cytoplasm (B), and 8 μm higher (C) at the level of the nucleus. Time is indicated in minutes, with time point 0 being the frame of nuclear envelope breakdown as judged by when tubulin dimers first enter the nucleus. Note increase in Ect2 levels in the cytoplasm before nuclear envelope breakdown in frames −1 and −2. Scale bars, 10 μm. (D) Quantification of time-lapse images in (B) and (C). Six cells were analyzed and measurements aligned, so that time point 0 represents the frame of nuclear envelope breakdown. Mean signal intensity was measured for Ect2 (red line) and tubulin (blue line) in a 6 × 6 pixel box in the nucleus and cytoplasm and the nuclear/cytoplasmic ratio was plotted. The black line shows mean cell length to give an indication of the onset of mitotic rounding. Error bars denote SD. See also Movie S2. See also Figure S4.

To understand how Ect2 might drive distinct changes in cell shape at the onset and exit of mitosis, we looked at its subcellular distribution during mitotic progression. In fixed cells stained with an Ect2 antibody, endogenous Ect2 was found to localize to the interphase nucleus and nucleolus (Figure 5A). At the onset of mitosis, Ect2 was then localized in the cytoplasm prior to nuclear envelope breakdown, where it remained until it was recruited to the spindle midzone at mitotic exit. We confirmed that this dynamic pattern of immunostaining was specific using Ect2 RNAi cells (Figure S4). Moreover, this localization was recapitulated using live imaging of a HeLa cell line constitutively expressing a BAC-containing mouse Ect2-GFP (Hutchins et al., 2010) (Figures 5B and 5C; Movie S2) and transfected with tubulin-RFP (Kobayashi and Murayama, 2009) as a marker of rounding (Figure 5B), nuclear envelope breakdown (visualized by the exclusion of tubulin from the nucleus; Figure 5C), and spindle morphogenesis. Ect2 could be seen accumulating in the cytoplasm in early prophase, ∼6 min before nuclear envelope breakdown and coincident with the onset of mitotic rounding (Figure 5D). In addition, we confirmed that the change in Ect2 localization at prophase was accompanied by the nuclear import of Cyclin B1, which has previously been shown to correlate with an increase in Cdk1 activity (Gavet and Pines, 2010a) and followed shortly after mitotic kinase substrate phosphorylation in the nucleus, which was detected using a phospho-Ser/Thr-Pro antibody (Figure S4). Ect2 contains two Cdk1 consensus sequences in the vicinity of its nuclear localization sequence (NLS). To test whether Cdk1 might act through these sites to regulate Ect2 localization, we generated a phospho-mimetic construct (Ect2-T342D-S366D). This localized to the interphase nucleus (Figure S4), suggesting that phosphorylation at these sites may not be sufficient to induce Ect2 nuclear export. However, this does not rule out the possibility that Cdk1 phosphorylation at other sites could control nuclear release.

### Mislocalization of Ect2 to the Cytoplasm Is Sufficient to Drive Premature Rounding

**Figure 6:**
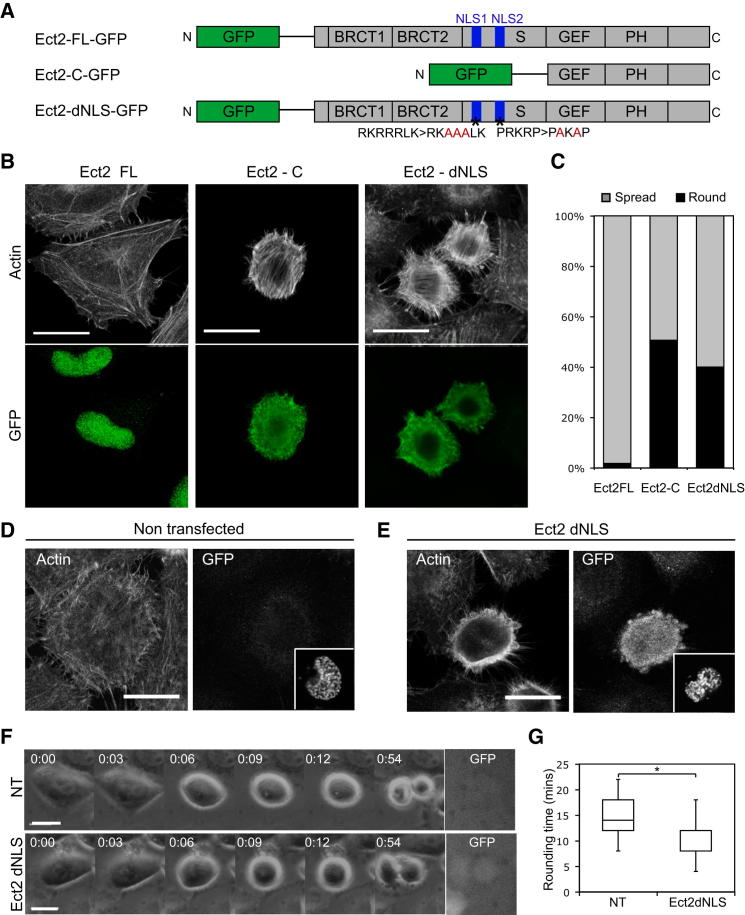
Cytoplasmic Ect2 Is Sufficient to Induce Cell Rounding (A) Three different Ect2 constructs were overexpressed in HeLa cells: Ect2-FL-GFP, Ect2-C-GFP, and Ect2-dNLS-GFP. (B) Representative confocal micrographs of cells transfected with Ect2-FL, Ect2-C and Ect2-dNLS showing the actin cytoskeleton stained with phalloidin-TRITC (top panel) and Ect2 construct localization (bottom panel). Note the rounded cell morphology in Ect2-C and Ect2-dNLS cells. (C) Quantification of the percentage of interphase cells displaying the rounded phenotype (n = 80–149 cells). (D and E) Representative confocal micrographs of cells in prophase showing an a nontransfected cell (D) and a cell transfected with Ect2-dNLS (E). The actin cytoskeleton was visualized by phalloidin staining and an anti-GFP antibody was used to indicate transfected cells. Inset shows the cell nucleus, stained with DAPI, to identify mitotic stage. Note rounded cell morphology in E. (F) Phase contrast images of a nontransfected (NT) cell and a cell expressing Ect2-dNLS-GFP at low levels rounding up in early mitosis. Transfected cells are indicated by GFP fluorescence in final panel. See also Movie S3. (G) Box plot comparing the mitotic rounding time of nontransfected cells (n = 21 cells) with those transfected with Ect2-dNLS-GFP (n = 23). To ensure rounding is mitotic rather than apoptotic, only cells that later proceeded to cytokinesis were analyzed. For Ect2-dNLS cells, only cells expressing low levels of the construct that were not already rounded in interphase were analyzed. For box plot, central line shows median, boxes are quartiles, and whiskers show range. For Ect2 dNLS, the median and lower quartile are the same value. Scale bars, 20 μm. See also Figure S5.

Since the recruitment of Ect2 to the spindle midzone triggers actomyosin-dependent furrow formation (Chalamalasetty et al., 2006; Nishimura and Yonemura, 2006; Yüce et al., 2005), we postulated that its relocation from the nucleus to the cytoplasm in prophase could be a key factor in driving mitotic rounding. We utilized several human Ect2 constructs to test this idea (Figure 6A). Mammalian Ect2 protein consists of an N-terminal BRCT repeat domain, which is the site of RacGAP1 binding, a regulatory S domain that harbors NLS sites, and a C-terminal catalytic GEF domain (Miki et al., 1993; Saito et al., 2004; Saito et al., 2003; Yüce et al., 2005). We confirmed first that when overexpressed, full length human Ect2 (Ect2-FL, (Niiya et al., 2006) is confined to the nucleus and does not affect cell morphology (Figure 6B). By contrast, a truncated form of Ect2 (Ect2-C, (Su et al., 2011), which contains the C-terminal catalytic domain but lacks regulatory regions and can act as a constitutively active form (Saito et al., 2004), is localized to the cytoplasm and is able to induce profound changes in interphase cell shape (Figure 6B). Approximately 50% of interphase cells expressing Ect2-C adopt a small, rounded morphology (Figures 6B and 6C; Figure S5). In this they resemble cells in mitosis, with the notable exception that they retain stress fibers, which are normally disassembled in prophase. Thus, the mislocalization of a constitutively active form of Ect2 is sufficient to induce ectopic rounding. As seen for mitotic cells, the interphase rounding induced by ectopic Ect2 is dependent on the activity of ROK and myosin II since it could be reversed by the addition of small molecular inhibitors Y-27632 or blebbistatin (Figure S5).

To test the role of nuclear export in the regulation of Ect2-dependent mitotic rounding, we introduced point mutations in its two NLS sites (Ect2 dNLS), which have been shown to prevent Ect2 nuclear import (Saito et al., 2004). Like Ect2-C, Ect2-dNLS was found to localize to the cytoplasm and to induce ectopic interphase rounding (Figures 6B and 6C). This demonstrates that simply mutating five residues in the NLS sequences is sufficient to induce gross changes in cell morphology. To test whether the timing of Ect2 export from the nucleus contributes to mitotic rounding we then arrested cells expressing low levels of Ect2-dNLS in G2 using the specific Cdk1 inhibitor, RO-3306 (Vassilev et al., 2006) for 14 hr. The inhibitor was washed out to enable cells to synchronously progress into mitosis in the presence or absence of Ect2-dNLS (Figures 6D and 6E). As expected, the presence of low levels of cytoplasmic Ect2-dNLS, just below the threshold required to cause interphase rounding, had a significant effect on the kinetics of rounding. Ect2-dNLS expressing cells already appeared quite spherical in prophase (Figure 6E) and underwent accelerated rounding upon entry into mitosis (Figure 6F and 6G; Movie S3). Thus, the appearance of cytoplasmic Ect2 is rate-limiting for mitotic rounding. Finally, we confirmed that both rounding and cytokinesis require the GEF activity of Ect2, since a full-length Ect2 construct containing a V566 > D mutation that has shown to be essential for GEF activity (van Impel et al., 2009) was unable to rescue either the failures in rounding or cytokinesis induced by Ect2 siRNA (Figure S5). These data suggest that Ect2 is active throughout mitosis, and that shifts in its localization regulate distinct changes in actomyosin organization and cell shape. These begin with the exit of Ect2 from the nucleus in early prophase, which functions as a key trigger for actomyosin remodeling as cells round up as they enter mitosis.

## Discussion

In this study we identify Ect2 as a critical link between the cell cycle machinery, which triggers numerous events that accompany mitotic entry, and the actin-dependent shape changes that occur in early mitosis. We show that Ect2 is able to induce changes to both cell shape and cortical mechanics in early mitosis through the activation of RhoA and remodeling of the actomyosin cytoskeleton. The timing of mitotic rounding is crucially dependent on the export of Ect2 from the nucleus in prophase, because cells lacking Ect2 fail to undergo timely mitotic rounding, whereas the mislocalization of Ect2 to the cytoplasm is sufficient, at low levels, to increase the speed of mitotic rounding and, at high levels, to induce ectopic mitotic-like rounding in interphase cells.

**Figure 7:**
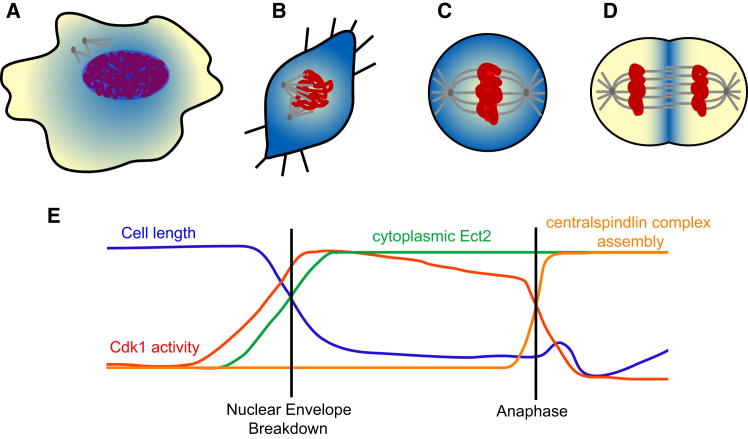
A Model for Ect2 Function through Mitosis (A–D) Dynamic changes in Ect2 localization (shown in blue) control actin remodeling throughout mitosis. (A) Ect2 leaves the nucleus in early prophase. (B) Active Ect2 in the cytoplasm is able to activate RhoA and drive mitotic rounding. (C) Ect2 activation of RhoA results in the formation of a rigid actomyosin cortex that assists metaphase spindle assembly (D). At anaphase, Ect2 is relocalized to the central spindle and removed from the poles, resulting in the redistribution of active RhoA and therefore the contractile actomyosin machinery to drive furrowing in the center of the cell. (E) Export of active, phosphorylated Ect2 into the cytoplasm at mitotic onset stimulates a decrease in cell length. At anaphase, Ect2 remains active but its location is modulated by binding to RacGAP1 at the spindle midzone, resulting in elongation of the cell, furrowing, and cytokinesis.

Together these data allow us to propose a model in which regulated changes in the localization of Ect2 drive stage-specific changes in mitotic cell shape (Figure 7). In prophase, Ect2 is exported from the nucleus and phosphorylated by Cdk1, which allow it to activate RhoA in the cytoplasm to induce the actomyosin reorganization and cell shape changes required for timely bipolar spindle assembly. It is likely that the resulting mechanically rigid metaphase cortex also plays an important role in buffering the spindle from the potentially disruptive influence of external mechanical forces (Kunda and Baum, 2009). At mitotic exit, it has been shown that Ect2 is recruited to the spindle midzone as the result of its binding to a core component of the centralspindlin complex, RacGAP1 (Burkard et al., 2009; Petronczki et al., 2007; Wolfe et al., 2009). There it repositions RhoA activity to control the formation of a circumferential actomyosin band across the center of the anaphase spindle (Chalamalasetty et al., 2006; Nishimura and Yonemura, 2006; Somers and Saint, 2003; Yüce et al., 2005), ensuring the precise segregation of contents between the two daughter cells. Thus, by driving stage-specific changes in the local activation of RhoA and the contractile actomyosin machinery, Ect2 is able to remodel mitotic cell shape; driving rounding in early mitosis and cleavage furrow formation at anaphase. Later, Ect2 is released from the spindle midzone soon after the onset of cytokinesis, leaving RacGAP1 free to catalyze mid-body maturation and abscission (Simon et al., 2008). The bulk of the protein is then degraded by the APC (Liot et al., 2011), while the remainder is reimported into the newly formed nuclei, to restore interphase cell shape. In this way, Ect2 resembles other mitotic proteins that display distinct roles at different times in mitosis, dependent on stage-specific changes in their localization e.g., Plk1 is known to associate with centrosomes at prophase, kinetochores at metaphase and the midzone at anaphase, enabling it to function in centrosome separation, microtubule attachment and cytokinesis respectively (Petronczki et al., 2008).

Ect2 has been shown to be phosphorylated on multiple sites by Cdk1 (Hara et al., 2006; Niiya et al., 2006; Su et al., 2011; Yüce et al., 2005). It is likely that this phosphorylation is required for its function in mitotic rounding, since Ect2 actively remodels the mitotic cortex in early mitosis under conditions of high Cdk1 activity. Previously, one Ect2 Cdk1-dependent phosphorylation site, T342, was shown to inhibit RacGAP1 binding and become dephosphorylated in anaphase (Yüce et al., 2005), leading to the speculation that Cdk1 phosphorylation could inhibit Ect2, functionally coupling the initiation of contractile ring formation to mitotic exit. Our data, however, argue that this is unlikely to be the sole mechanism by which Ect2 is regulated, since we see little change in the global Ect2 phosphorylation level at anaphase and it remains in a hyper-phosphorylated state through until the end of cytokinesis (Figure 4). In line with this, Ect2 phosphorylation has been shown to relieve an auto-inhibitory interaction between the C- and N-terminal domains of the protein (Hara et al., 2006; Kim et al., 2005) and to be essential for its GEF activity (Tatsumoto et al., 1999). These data suggest that Cdk1 phosphorylation contributes to the activation of Ect2 at anaphase. A recent study identified a role for the C terminus of Ect2 in its recruitment to the membrane at anaphase (Su et al., 2011). This membrane localization was shown to be essential for cytokinesis. Interestingly it was also suggested that this change in localization at anaphase may be triggered by a change in CDK-mediated phosphorylation at T815. It is clear from our analysis, however, that Ect2 is able to activate RhoA at the membrane in metaphase despite its having a largely diffuse cytoplasmic localization. Thus, the dephosphorylation of Ect2 at this site at anaphase likely induces a change in the rates at which Ect2 shuttles between the membrane and cytoplasm, and may function to limit the range of Ect2′s action to allow for polar relaxation (Sedzinski et al., 2011).

If Cdk1-mediated phosphorylation of Ect2 plays a role in the regulation of its activity at both metaphase and anaphase, an important unsolved question is how bulk Ect2 phosphorylation persists following the inactivation of Cdk1/CyclinB at mitotic exit. This may be the result of Plk1-dependent phosphorylation of Ect2 at the midzone (Niiya et al., 2006) or the result of the dynamic regulation of Ect2 dephosphorylation by mitotic phosphatases (Barr et al., 2011; Bouchoux and Uhlmann, 2011). In addition, there may be subtle changes in the set of Ect2 phosphorylation sites that accompany mitotic progression which change the relative potency of Ect2 and/or its specificity to tune its RhoGEF activity to the generation of a rounded cortex during mitotic entry or an actomyosin ring at mitotic exit (Su et al., 2011; Yüce et al., 2005). A comprehensive dissection of the function and dynamics of Ect2 phosphorylation through mitosis however is likely to remain a challenge for some time as it is hampered by the sheer number of sites revealed in both biochemical studies (Hara et al., 2006; Niiya et al., 2006; Yüce et al., 2005) and large-scale screens for mitotic phosphorylation (Beausoleil et al., 2006; Dephoure et al., 2008).

Although Ect2 provides a critical link between mitotic entry and cell rounding, it is clear that other factors are important in the control of mitotic cell shape. These include the loss of substrate adhesion, which is dependent on Rap1 inhibition (Dao et al., 2009) together with changes in osmotic pressure (Stewart et al., 2011). This may explain why Ect2-depleted cells, although suffering from profound defects in actin organization, eventually assume a roughly rounded morphology (Figure 1). Indeed, we observed no defects in the timing of focal adhesion disassembly in Ect2 RNAi cells (Figure S3), suggesting that loss of adhesion may allow cells to decrease in length despite defects in actin organization and myosin contractibility. It is therefore likely that Cdk1/CyclinB and other mitotic kinases directly regulate focal adhesion removal and changes to ion channels in parallel, independently of Ect2, to ensure their coordination. Nevertheless, the involvement of Ect2 in sequential events during mitosis suggests that it is a central organizer of the cortex through mitotic progression. Significant changes to the actomyosin cytoskeleton occur at rounding when actin filaments are rearranged to form a stiff cortical shell (Kunda et al., 2008) and then at cytokinesis when the symmetry is broken by furrowing and polar relaxation (Eggert et al., 2006). Since our data suggest that both processes are controlled by the same molecular “toolbox,” downstream of Ect2 and RhoA, one might speculate that they are mechanistically coupled. Thus, the repositioning of Ect2 at anaphase, may serve to loosen the rigid actomyosin cortex at the cell poles, coupling cleavage furrow formation to polar relaxation. In fact, this was long the favored model for cytokinesis, in which polar relaxation was through to precede and to drive furrow formation (Roberts, 1961).

There are several similar parallels in evolution where actin remodeling events required for cytokinesis are initiated before cell division. In the early *Caenorhabditis elegans* embryo, cortical actomyosin flows determine cell polarity before division, in a process that is dependent on Ect2 and RhoA (Motegi and Sugimoto, 2006; Schonegg and Hyman, 2006). Likewise in fission yeast, preparation for cytokinesis begins before anaphase with the formation of actin-nucleating nodes at the onset of mitosis, which later condense to form the contractile ring (Goyal et al., 2011; Pollard and Wu, 2010). Our data suggest that a similar mechanism could operate in mammalian cells, with the events that remodel the actin cortex in preparation for cell division being initiated by Cdk1-dependent Ect2 activity at the onset of mitosis, rather than by Cdk1 inhibition at anaphase.

## Experimental Procedures

### Time-Lapse Microscopy

For live imaging, HeLa stable cell lines expressing LifeAct-GFP/histone2B-mRFP, histone2B-mRFP/tubulin-GFP (Steigemann et al., 2009), Ect2-GFP (hela Kyoto -mEct2-GFP-FLAP (Poser et al., 2008; Hutchins et al., 2010), and a tetracycline-inducible line expressing Cyclin-B1-Venus (Di Fiore and Pines, 2010) were plated on glass-bottomed dishes (MatTek) coated with 10 μg/ml fibronectin (Sigma). For cell length measurements, cells were imaged every 2 min using a Zeiss Axiovert 200M microscope with a 20× objective (numerical aperture, NA 0.4), and images acquired using a Hamamatsu Orca AG camera and Volocity software (Perkin Elmer). Cell length was defined as the furthest distance between two points on the cell perimeter (Feret's Diameter) and measured using Fiji. For filming inhibitor treated cells, inhibitors were dissolved in DMEM + 10% FBS and added one hour prior to commencing filming, except for C3 transferase, which was added 6 hr before filming began. Inhibitors were used at the following concentrations: 100 ng/ml nocodozole (Sigma), 2 μg/ml C3 transferase (Cytoskeleton), 50 μM Y-27632 (Calbiochem), and 50 μM blebbistatin (Sigma). For time-lapse confocal imaging, an UltraView Vox (Perkin Elmer) spinning disc system was used with 60× oil immersion objective (NA 1.4). Images were acquired every 30 s with z slices every 4 μm covering the height of the cell. Single z plane images are shown.

### Immunofluorescence

For immunostaining, cells were plated on fibronectin-coated glass coverslips and fixed with 4% formaldehyde for 20 min, permeablized with 0.2% triton-X in PBS for 5 min, blocked with 5% bovine serum albumin in PBS for 30 min and treated with primary and secondary antibodies for 1 hr at room temperature. Primary antibodies were used at the following dilutions: tubulin 1:400 (DM1A, Sigma-Aldrich), Ect2 1:200 (sc-1005 Santa Cruz), anti-GFP 1:200 (a11122 Molecular Probes), anti-myc 1:500 (9E10 Santa Cruz), phospho-Myosin Light Chain (T18/S19) 1:100 (Cell Signaling Technology 3674), phospho-Ezrin (T567)/ Radixin (T564)/ Moesin (T558) 1:200 (Cell Signaling Technology 3141), phospho-paxillin 1:200 (BD Bioscience 610051), and phospho-Ser/Thr-Pro 1:500 (MPM2, Millipore). Secondary anti-rabbit IgG and anti-mouse IgG antibodies (Molecular Probes) tagged with alexa-fluor 488, 546 or 647 were used at 1:500. TRITC-conjugated phalloidin (Sigma) was used at 0.1 μg/ml and DAPI (Invitrogen) at 1:1000. Immuno-stained cells were mounted with FluorSave (Calbiochem) and imaged on a Leica SPE confocal microscope with a 63× lens (NA 1.3).

### Gel Mobility Shift Assay and Western Blotting

Ect2 bandshift experiments were carried out as previously described (Niiya et al., 2006; Tatsumoto et al., 1999). Unsynchronized cells or cells arrested in mitosis by 5 μM STLC (Sigma) treatment for 18 hr were harvested in Laemmli Buffer (Sigma). Roscovitine (Calbiochem) was used at a concentration of 50 μM for 2 hr. For block and release experiments, cells were synchronized by treatment with 2 mM thymidine (Sigma) for 14 hr, released into fresh medium for 8 hr, followed by a second 14 hr 2 mM thymidine treatment, and then released for 10 hr before harvesting every hour. For mitotic exit, synchronization cells were synchronized at metaphase using the following protocol: 24 hr treatment with 2 mM thymidine, followed by 6 hr release, 4 hr in 100ng/ml nocodozole, and finally 2 hr in 10 μM Mg132. Cells were then released into fresh media and lysed at 20 min intervals. Samples were loaded onto an 8% SDS-PAGE gel before transfer onto an Immobilon-P (Millipore) membrane by western blotting. Membranes were blocked in 5% BSA in TBST for 1 hr, incubated overnight at 4°C with primary antibodies, and for several hours at room temperature with secondary antibodies. Antibodies were used at the following dilutions: Ect2 1:500 (sc-1005 Santa Cruz), Cyclin B1 1:500 (sc-595 Santa Cruz), adaptin γ 1:2000 (BD Biosciences 610386), phospho-histone H3 (S10) 1:4000(Cell Signaling Technology 9706), and tubulin 1:2000 (DM1A, Sigma) and HRP-conjugated secondary antibodies 1:1000 (DAKO). Results were visualized using an ImageQuant LAS4000 system and blots quantified using ImageQuant TL software (GE Healthcare).

### Optical Stretching

Cells were plated at 90% confluency in 25 cm^2^ flasks for RNAi treatment. Three hours after RNAi treatment, cells were arrested in G2 by addition of 9 μM RO-3306 (Enzo Life Science) for 18 hr, before release from the drug for 90 min and harvest by mitotic shake-off. Interphase cells and multinucleate Ect2 RNAi cells that had already failed cell division were excluded from the analysis by visual inspection of DNA and measurement of cell diameter inside the optical stretcher. The microfluidic optical stretcher (OS) has previously been described in detail (Lincoln et al., 2007). Two counter-propagating near-infrared laser beams (γ = 1064 nm), emerging from single-mode optical fibers, are combined in a microfluidic channel to trap (low power p = 0.1 W/fiber) and deform (p = 1.0 W/fiber) single suspended cell by optically induced surface stresses. Cells were introduced into the OS at a concentration of 50 × 10^4^ cells/ml and experiments were done at room temperature (22°C). The mechanical properties of cells were measured by a creep compliance experiment. This test enables a direct comparison between the mechanical properties of cells (Lautenschläger et al., 2009; Wottawah et al., 2005). A cell is first held at low power in the optical trap and then a constant stress is applied to the cell for 4 s (p = 1W). The relative cell deformation, or strain *D*(*t*) = Δ*r*(*t*)/*r*_o_ (during and after application of the stress) is recorded with phase contrast videomicroscopy. The compliance of the cell, representing a true material property, is defined as *J*(*t*) = *D*(*t*)/(σ_o_ *F*_G_), which is the strain *D*(*t*) normalized by the peak stress applied, σ_o_, and a geometrical factor *F*_G_ (Ananthakrishnan et al., 2006; Guck et al., 2001), which accounts for different cell sizes or different refractive indices. The refractive indices of cells were determined by immersion refractometry using BSA solutions as described previously (Guck et al., 2005): *n*_mitotis_ = 1.3510 ± 0.0096 and *n*_interphase_ = 1.3563 ± 0.0041.

### FRET Analysis

HeLa cells were transfected with a RhoA YFP-CFP FRET biosensor (Pertz et al., 2006) and 32 hr after transfection 5 μM STLC was added for 15 hr to synchronize cell in mitosis. Cells were then fixed with 4% formaldehyde and imaged using a Leica SP5 scanning confocal system with a 63x oil objective (NA 1.4). FRET efficiency was calculated using acceptor photobleaching as described (Matthews et al., 2008). Briefly, CFP and YFP channels were excited using the 458 nm and 514 nm lasers respectively. Cells were imaged prebleach, then a region consisting of half the cell was bleached for 2 min using the 514 nm laser at maximum power. Postbleach images were then acquired for each channel and the total FRET efficiency ratio for the bleached half of the cell was calculated as (CFP_postbleach_ − CFP_prebleach_)/CFP_postbleach_. An efficiency ratio was also calculated for an equal-seized nonbleached region and subtracted from the bleached region to give the final ratio.

### Statistical Analysis

Graphs were produced and statistical analysis carried out in Microsoft Excel. Bar charts show mean values with error bars representing standard deviation. Box plots show median as line, upper and lower quartiles as box, and range as whiskers. The p values were calculated using the student's t test (two sample equal variance, two-tailed), ^∗^p < 0.01, ^∗∗^p < 0.001.

## Supplemental Information

Document S1. Figures S1–S5, Table S1, and Supplemental Experimental Procedures

Movie S1. HeLa Cells Treated with Control siRNA and Ect2 siRNA and Labelled with LiveAct-eGFP and Histone H2B-mcherry Round Up as They Enter Mitosis, Related to Figures 2A and 2B

Movie S2. HeLa Cell Expressing Mouse-Ect2-GFP and Tubulin-mRFP Rounds Up and Enters Mitosis, Related to Figures 5B and 5C

Movie S3. Phase Contrast and GFP Fluorescence Movies of a Nontransfected HeLa Cell and a Cell Transfected with Ect2-dNLS-GFP Rounding Up as They Progress through Mitosis, Related to Figure 6F
