# Supplementary material for: Changes in Ect2 Localization Couple Actomyosin-Dependent Cell Shape Changes to Mitotic Progression
Source: Dev Cell. 2012 Aug 14;23(2):371–83. doi: 10.1016/j.devcel.2012.06.003 (PMC3763371; doi:10.1016/j.devcel.2012.06.003)
Supplement: Document S1. Figures S1–S5, Table S1, and Supplemental Experimental Procedures [file mmc1.pdf]

Developmental Cell, Volume *23*

Supplemental Information

Changes in Ect2 Localization

Couple Actomyosin-Dependent Cell Shape

Changes to Mitotic Progression

Helen K. Matthews, Ulysse Delabre, Jennifer L. Rohn, Jochen Guck, Patricia Kunda, and Buzz Baum

INVENTORY OF SUPPLEMENTAL INFORMATION

SUPPLEMENTAL DATA

Figure S1, related to Figure 1. Ect2 is required for mitotic rounding in other cell types.

Figure S2, related to Figure 2. Mitotic cells are less compliant than interphase cells and Ect2 is required for timely bipolar spindle assembly.

Figure S3, related to Figure 3. Ect2 affects myosin activity at mitosis but not focal adhesions or ERM activity.

Figure S4, related to Figure 5. Ect2 leaves the nucleus when nuclear CyclinB1/Cdk1 activity accumulates.

Figure S5, related to Figure 6. Ect2 construct over-expression experiments.

Table S1. List of genes included in RNAi screen for mitotic rounding and main hits.

SUPPLEMENTAL EXPERIMENTAL PROCEDURES

SUPPLEMENTAL REFERENCES

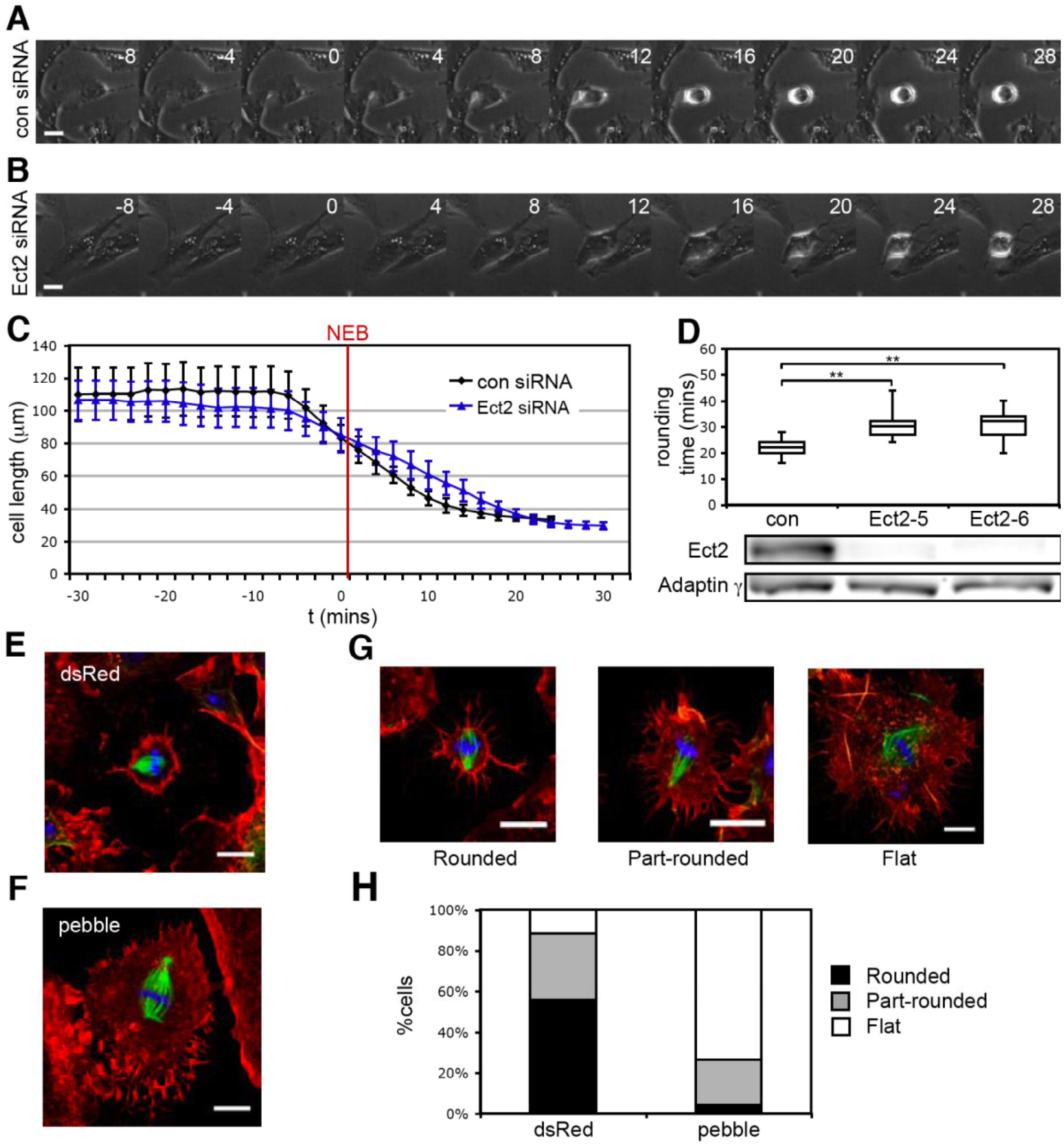

Figure S1, related to Figure 1. Ect2 is required for mitotic rounding in other cell types.

(A-D) Ect2 is required for timely mitotic rounding in RPE1 cells. (A, B) Phase contrast stills from a timelapse video of RPE1 cells rounding up to enter mitosis showing cells treated with control siRNA (A) and Ect2 siRNA (B). (C) Graph showing the mean cell length for control and Ect2 siRNA cells entering mitosis, aligned so that timepoint 0 is the frame of nuclear envelope breakdown. Error bars denote S.D. (D) Box plot comparing rounding times for RPE1 cells treated with control (*n* = 15) and two different non-overlapping siRNAs against Ect2 (Ect2-5, *n* = 19 and Ect2-6, *n* = 19). Central line shows mean, box is quartiles and whiskers show complete range. Lower panel shows western blot to demonstrate the efficiency of knockdown by both siRNAs. Note that both siRNAs significantly delay rounding in RPE1 cells as in HeLa cells.

(E-H) The fly homologue of Ect2, *pebble*, is required for mitotic cell rounding in the S2R+ *Drosophila* cell line. (E, F) Confocal micrographs of S2R+ cells in metaphase in (E) a cell

treated with dsRed dsRNA as a control and (F) dsRNA to knock down *pebble*. Actin is visualised with phalloidin in red, tubulin showing the spindle in green and DNA stained with DAPI in blue. Note the change in cell size, morphology and actin organisation in the *pebble* knockdown. Cortical actin is removed and the cell remains flat in mitosis. (G) Representative images showing three phenotypes observed at metaphase in S2R+ cells: rounded cells with cortical actin, part-rounded cells that have retracted their margin but not formed an actin cortex and cells that remain completely flat at mitosis. (H) Quantification of the percentage of cells with each phenotype for dsRed (n = 43) and *pebble* knockdown (n = 47). Knockdown of *pebble* results in significant disruption to mitotic rounding. Scale bars 10 μm.

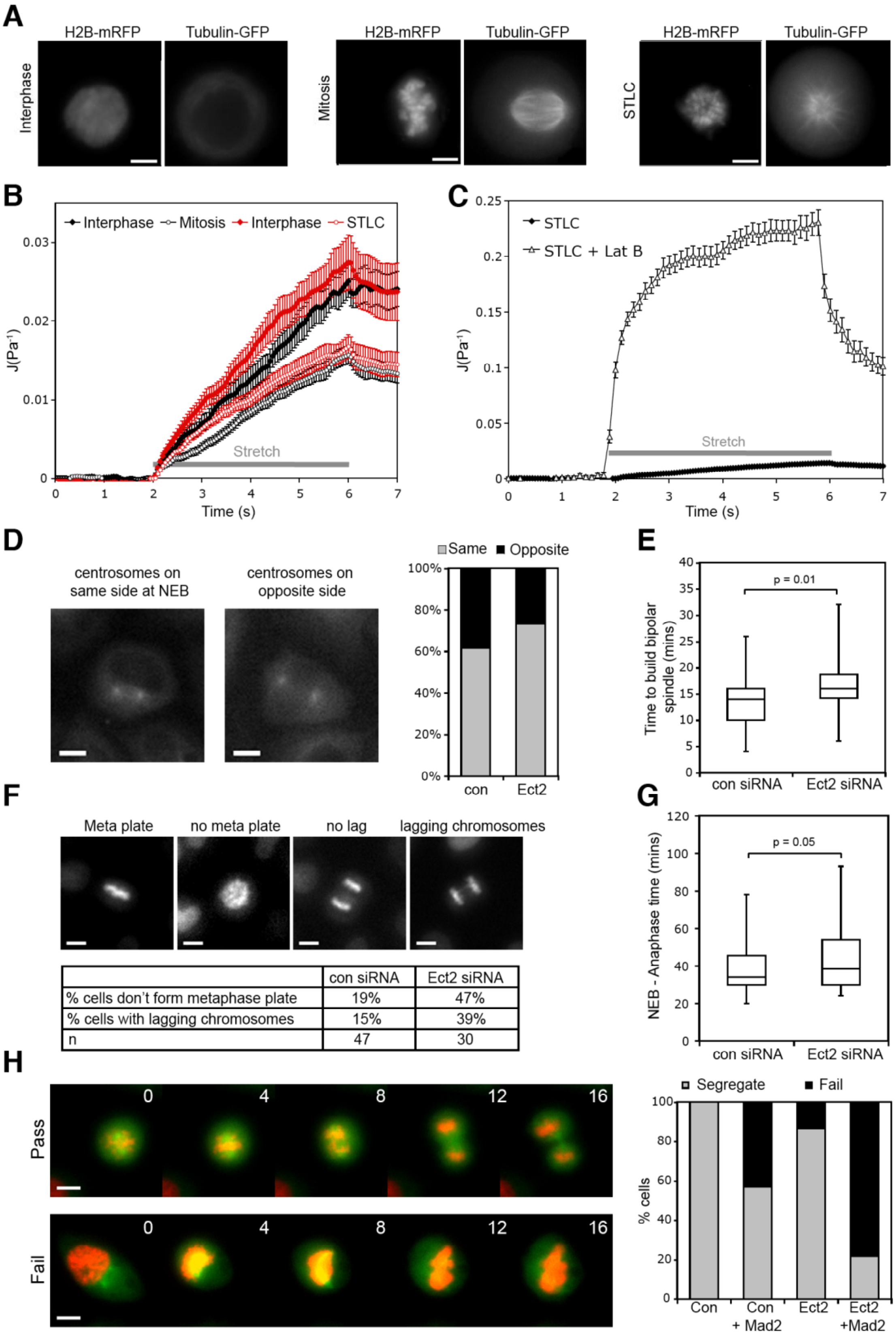

	con siRNA	Ect2 siRNA
% cells don't form metaphase plate	19%	47%
% cells with lagging chromosomes	15%	39%
n	47	30

Figure S2, related to Figure 2. Mitotic cells are less compliant than interphase cells and Ect2 is required for timely bipolar spindle assembly.
(A-C) Mitotic cells are less compliant than interphase cells, in an actin-dependent manner. (A) Fluorescence images of HeLa cells in the optical stretcher, used to characterise cells as interphase and mitotic by imaging DNA labelled with histone-2B Cherry and the spindle labelled with tubulin-GFP. (B) Graph showing the mean compliance (see experimental procedures) of HeLa cells during a four second stretch. Red closed diamonds show unsynchronised cells (*n* = 22 cells), open red diamonds show cells synchronised in prometaphase by treatment with 5 μM STLC for 15h (*n* = 39). Black lines show cells synchronised in G2 by 18h treatment with 9 μM RO-3306 and released into drug free media for 90 min, in interphase (closed circles, *n* = 16) compared to mitosis (open circles, *n* = 60). Interphase or mitotic cells were identified by visual inspection. Note that mitotic cells are less compliant than interphase cells. There was no difference in compliance between STLC cells and mitotic cells with a bipolar spindle, suggesting that the spindle does not greatly affect cell mechanics. (C) Graph showing the mean compliance of HeLa cells during a four second stretch comparing cells arrested in mitosis using STLC (closed diamonds, *n* = 46) with STLC-arrested cells treated with 5 μM Latrunculin B to depolymerise actin filaments immediately before stretching (open triangles, *n* = 27). Cell lacking f-actin are extremely compliant compared to untreated cells. Error bars denote S.E.M. Scale bars 5 μm.
(D-H) Ect2 is required for timely bipolar spindle assembly in HeLa cells.(D-G) HeLa cells expressing Histone H2B-mCherry and Tubulin-GFP was filmed every 2 minutes and a number of defects in mitotic spindle assembly were observed in Ect2 RNAi treated cells. (D) Ect2 siRNA cells have delayed centrosome separation. Cells were classified by whether their centrosomes were on the same side (left panel) or opposite sides (right panel) of the nucleus at the frame before nuclear envelope breakdown (NEB). Graph shows that a higher proportion of Ect2 siRNA cells (*n* = 30 cells) have centrosomes at the same side at NEB compared to controls (*n* = 47). (E) Graph showing time taken to assemble a bipolar spindle in control siRNA cells (*n* = 47) compared to Ect2 siRNA cells (*n* = 30). This may reflect the delay in centrosome separation shown in D. For box plots, central line is median, box is quartiles and whiskers show range. (F) Images showing defects observed in chromosomal organisation in Ect2 siRNA cells with quantification of the percentage of cells affected. Defects in chromosome congression to the metaphase plate as well as metaphase plate misalignment (left panel, 'no meta plate') were present at metaphase, while at anaphase, lagging chromosomes were often observed (right panel). (G) The defects in spindle assembly shown in D-F translated into a significant delay to anaphase onset time in Ect2 RNAi cells, likely through activation of the spindle assembly checkpoint. Box plot shows time from nuclear envelope breakdown to anaphase onset in control siRNA cells compared to Ect2 siRNA cells. (H) Experiment showing the effect of perturbing the spindle assembly checkpoint using Mad2 siRNA depletion, which forces cells to rapidly undergo anaphase in under 20 minutes after NEB. Under these conditions, many cells are still able to build a bipolar spindle and segregate their chromosomes, albeit messily (example in top panel, 'pass') while others are unable to build a spindle in time and exit mitosis with only one nucleus (example in bottom panel, 'fail'). Graph shows the percentage of cells that are able to segregate their chromosomes in cells treated with control siRNA (*n* = 35), Mad2 siRNA alone (*n* = 21), Ect2 siRNA alone (*n* = 27) and Ect2 and Mad2 siRNAs combined (*n* = 32). Note that the majority of cells are able to segregate their chromosomes in Mad2 siRNA alone but this number is dramatically decreased when Mad2 siRNA is combined with Ect2 siRNA, reflecting the delay in bipolar spindle assembly in these cells. Scale bars, 10 μm.

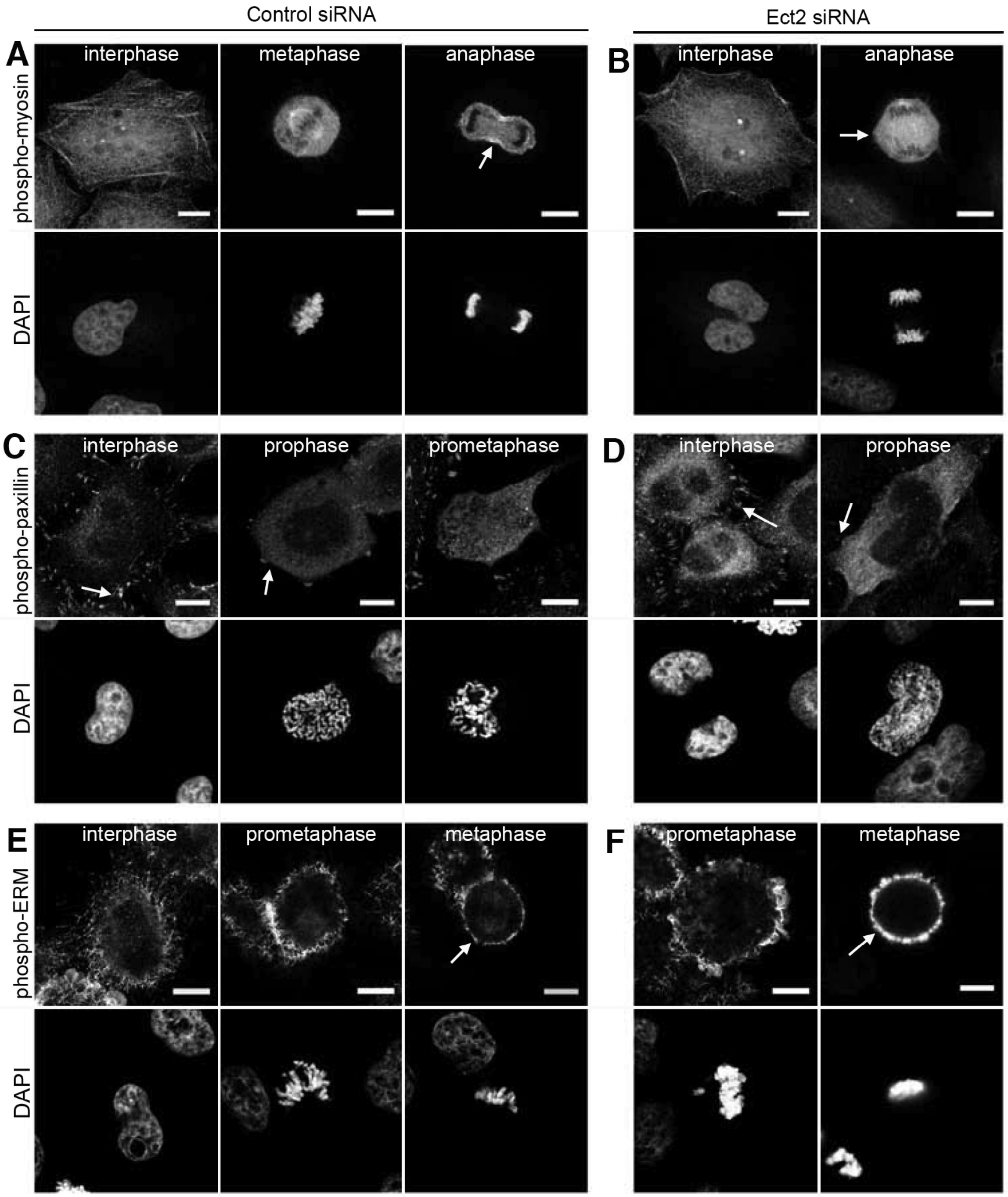

Figure S3, related to Figure 3. Ect2 affects myosin activity at mitosis but not focal adhesions or ERM activity.

(A, B) Confocal images of control and Ect2 RNAi cells stained with phospho-myosin (T18/S19) regulatory light chain (pMyo) antibody to show myosin activity and DAPI to show DNA. (A) In control cells the pMyo antibody localises to stress fibres in interphase but is removed from stress fibres at prophase where it can be seen at the cell edge during cell rounding (Figure 3F). No specific localisation can be observed in round metaphase cells but pMyo localises to the furrow at anaphase. (B) Ect2 siRNA cells also display pMyo-rich stress fibres at interphase, but a decrease in myosin activity in mitosis

is observed with no myosin observed at the cell margin during rounding (Figure 3G) or at the furrow at anaphase (arrow). (C, D) Confocal micrographs of control (C) and Ect2 siRNA (D) cells stained with a phospho-paxillin antibody to show focal adhesions and DAPI to show DNA. Both control and Ect2 RNAi cells show a similar pattern of staining with many punctuate focal adhesions in interphase (arrows), which disassemble in early prophase. (E, F) Confocal images showing cells stained with a phospho-Ezrin/moesin/radixin (pERM) antibody and DAPI at different mitotic stages. Control HeLa cells (E) have punctuate pERM activity covering the cortex in interphase, which increases in mitosis where pERM activity is high all around the cortex (arrow). (F) Ect2 siRNA cells show similarly high levels of ERM activity at the cortex in mitosis (arrow). Scale bars, 10 μm.

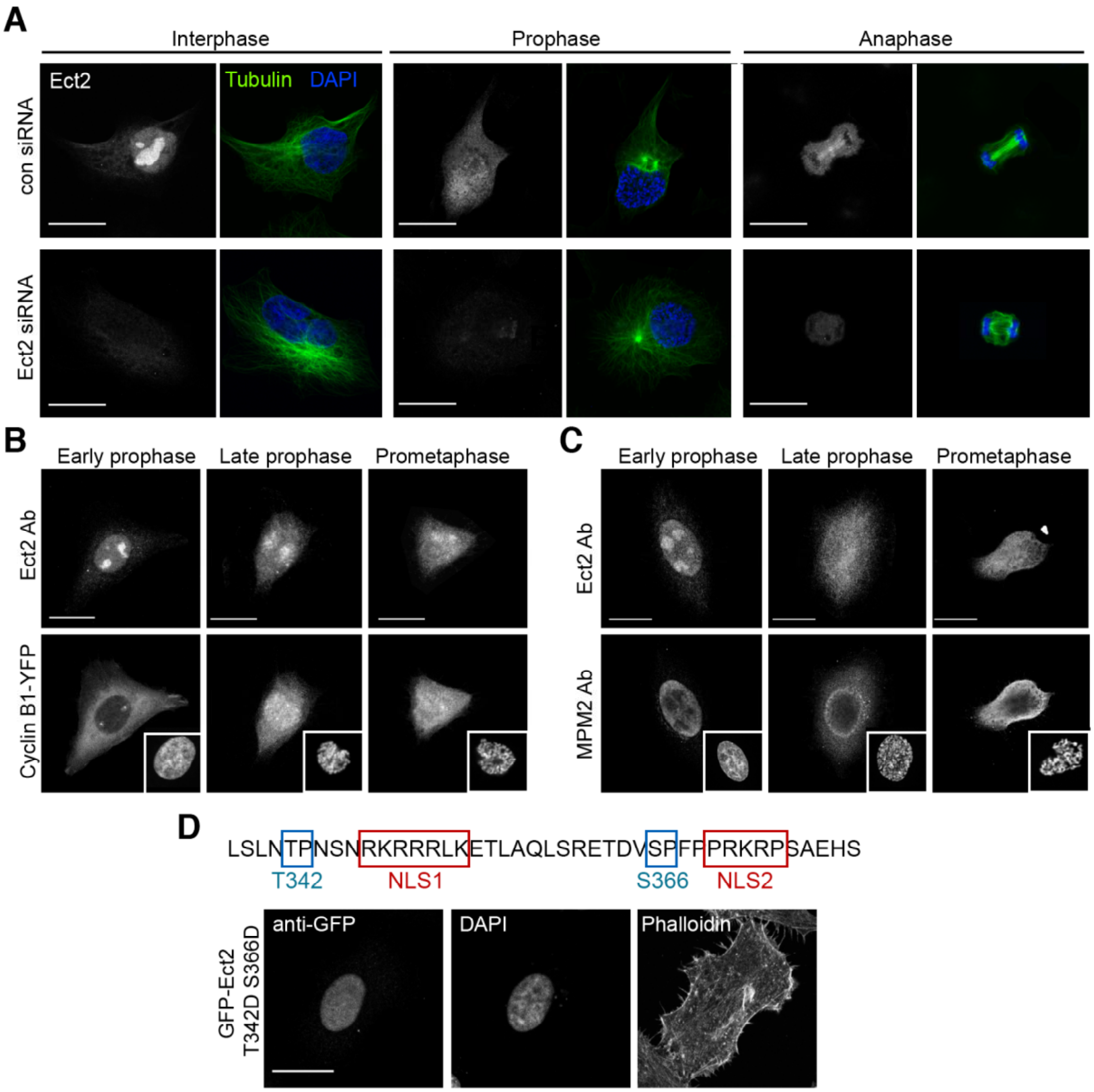

Figure S4, related to Figure 5. Ect2 leaves the nucleus when nuclear CyclinB1/Cdk1 activity accumulates.

(A) The Ect2 antibody staining is specific. To demonstrate antibody specificity, Ect2 was knocked down using siRNA (bottom panel) and compared to control siRNA (top panel). Ect2 is shown in grey, while mitotic stage is visualised using Tubulin-FITC (green) to mark microtubules and DAPI (blue) to stain DNA. Note that Ect2 siRNA abolishes the interphase nuclear signal, mitotic cytoplasmic signal as well as the anaphase spindle midzone localisation.

(B) Ect2 leaves the nucleus when cyclin B1 is imported into the nucleus. A stable cell line expressing Cyclin B1-YFP (shown in bottom panel) was fixed and stained with the antibody against Ect2 (top) and DAPI (inset). Import of cyclin B1 into the nucleus occurs in prophase and is known to mark an increase in nuclear Cdk1 activity. Here, in early prophase, Cyclin B1 is cytoplasmic while Ect2 is still confined to the nucleus. In late prophase, Cyclin B1 appears in the nucleus and now Ect2 has been released from the

nucleus and is visible in the cytoplasm. After NEB, at prometaphase both proteins freely localise to the cytoplasm.
(C) Mitotic kinase activity can be visualised in the nucleus before Ect2 is exported. As another readout of nuclear Cdk1 activity, an antibody that recognises phosphorylated mitotic kinase consensus sites (MPM2, lower panel) was used and compared to the localisation pattern of the Ect2 antibody (upper panel). Inset show DNA stained with DAPI. MPM2 staining can first be observed in the early prophase nucleus, when Ect2 is also nuclear. Nuclear MPM2 staining persists into late prophase by which time Ect2 is cytoplasmic. This indicates that Cdk1 is likely active in the nucleus before Ect2 is exported and therefore could potentially be playing a role, directly or indirectly in its nuclear export.
(D) Ect2 nuclear localisation is unlikely to be regulated by direct Cdk1 phosphorylation at the NLS. We identified 2 Cdk1 consensus sites (T342 and S366), close to the two NLS regions in the Ect2 protein sequence and hypothesized that phosphorylation and masking of these sites could be a mechanism by which Cdk1 activity could induce Ect2 nuclear export. However an Ect2-GFP construct containing phospho-mimetic mutations at both sites (Ect2-T342DS366D, left hand panel) was still able to localise to the nucleus (middle panel), suggesting that phosphorylation at these sites is unlikely to be sufficient to affect Ect2 nuclear localisation. Scale bars, 20 μm.

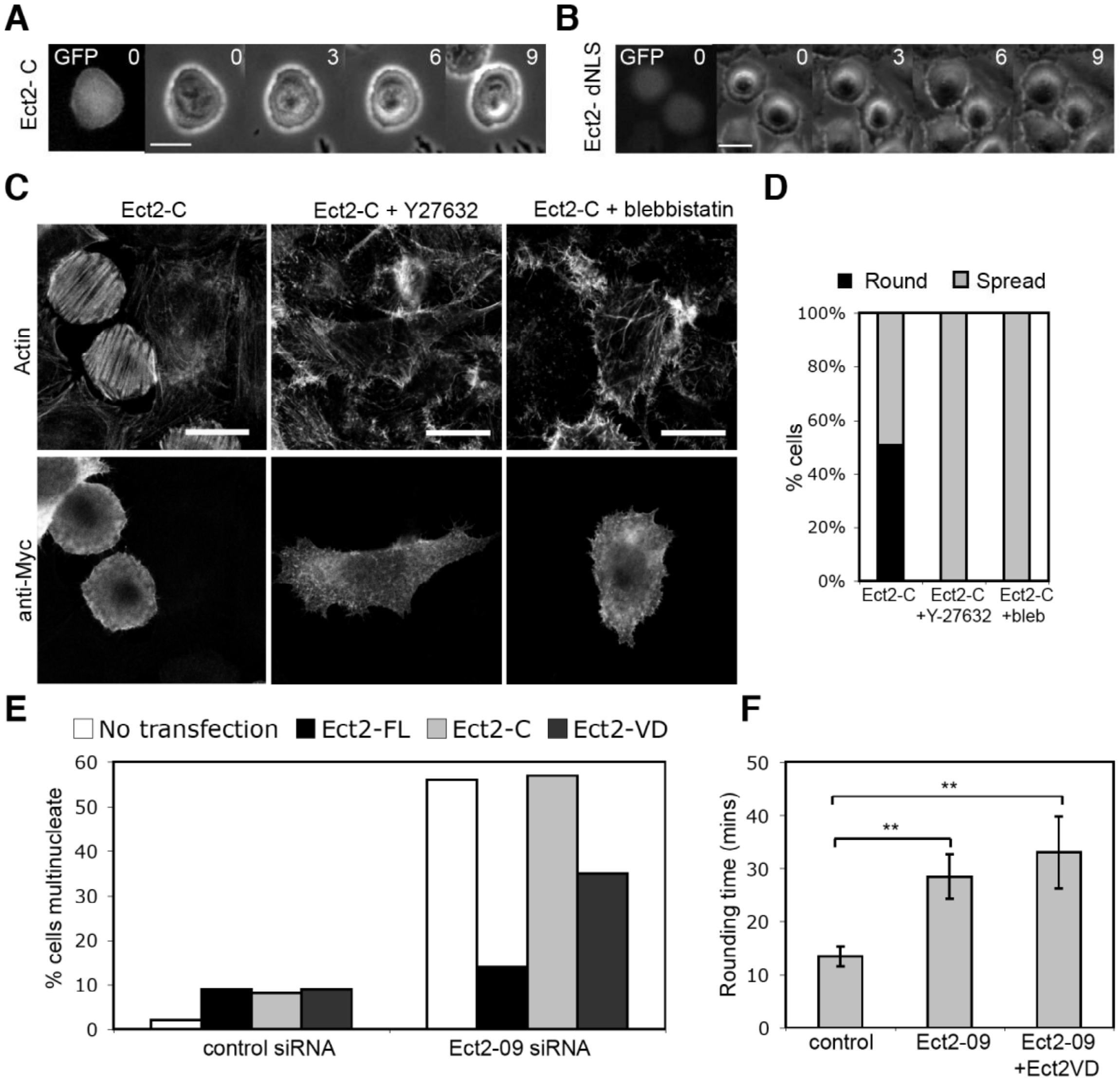

Figure S5, related to Figure 6. Ect2 construct over-expression experiments.
(A-B) HeLa cells are able to tolerate over-expression of Ect2-C-GFP construct (A) and Ect2 dNLS-GFP (B) for many hours without undergoing apoptosis and while maintaining a rounded interphase morphology. Phase contrast images show rounded morphology while GFP images (first panel) indicate transfected cells over a 9 hour time frame. During this time images were acquired every 2 minutes, time shown is in hours.
(C-D) The rounded interphase morphology induced by Ect2-C is dependent on signalling through Rho kinase and Myosin II. (C) Confocal micrographs showing HeLa cells transfected with Ect2-C with actin stained with phalloidin and transfected cells visualised with an anti-myc antibody. Ect2-C cells show a characteristic round morphology in interphase, which is abolished by treatment with 50 μM Y-27632 to inhibit Rho kinase or 50 μM blebbistatin to inhibit Myosin II contractility for 2 hours. (D) Quantification of percentage of cells showing the round morphology in the different conditions in C (*n* = 163, 28 and 31).
(E-F) The GEF activity of Ect2 is required for its roles in both mitotic rounding and cytokinesis. (E) Experiment testing the ability of different Ect2 over-expression

constructs to rescue the failure in cytokinesis brought about by Ect2 siRNA treatment. Cells were treated with Ect2 siRNA for 72 hours and constructs were transfected 15 hours after RNAi. The Ect2-09 siRNA was used, as it targets the 3' untranslated region of Ect2 mRNA and therefore does not target the over-expressed human constructs. Cells were then fixed and stained for Tubulin and DAPI and the number of multinucleate cells that had failed cytokinesis was counted (*n* = 68 -257 cells for each condition). Note that full length Ect2 is able to rescue the effect of Ect2 RNAi, but the C terminus alone (Ect2-C), or a GEF-dead version of Ect2 (V566>D, Ect2-VD) is not. (F) Experiment assessing the ability of GEF-dead Ect2 (Ect2-VD) to rescue the delay in mitotic rounding caused by Ect2 knockdown. Cells were transfected with the plasmid DNA constructs, then after 24 hours treated with Ect2 RNAi and after a further 24 hours were filmed and the duration of rounding measured. As before, Ect2-09 delays rounding (*n* = 10 cells) compared to a control siRNA (*n* = 8) and expression of GEF-dead Ect2 is unable to rescue this phenotype (*n* = 9) unlike full length Ect2 (see Figure 1E). Graph shows mean and error bars denote standard deviation. Scale bars 20 μm.

Gene	*Function*	*Hit?*	*Gene*	*Function*	*Hit?*
ABI1	WAVE complex		FMN2	Formin	
ABI2	WAVE complex		FMNL1	Formin	
ACTR2	ARP2/3 complex		FMNL2	Formin	
ACTR3	ARP2/3 complex		FMNL3	Formin	
ANLN	Actin binding protein		GSN	Filament severing	
CAP1	Actin binding protein		**INCENP**	Centromere protein	
CAP2	Actin binding protein		INF2	Formin	
**CAPZA1**	Filament capping		MYOH10	Myosin heavy chain	
CAPZA2	Filament capping		MYL9	Myosin light chain	
CAPZA3	Filament capping		MYO10	Unconventional myosin	
CAPZB	Filament capping	Y	NCKAP1	WAVE complex	
**CDC42**	Rho GTPase		PAK1	Rho GTPase effector	
**CFL1**	Filament severing		PAK3	Rho GTPase effector	
CFL2	Filament severing		**PALLD**	Filament organisation	
CORO1C	Actin binding protein		RAC1	Rho GTPase	
**CTTN**	Actin binding		RAC2	Rho GTPase	
CYFIP2	WAVE complex		RHOA	Rho GTPase	
DAAM1	Formin		RHOB	Rho GTPase	
DAAM2	Formin		RHOC	Rho GTPase	
**DIAPH1**	Formin		ROCK1	Rho Kinase	
DIAPH2	Formin		**ROCK2**	Rho Kinase	
DIAPH3	Formin		SPIRE1	Filament nucleation	
DSTN	Filament disassembly		SPIRE2	Filament nucleation	
**ECT2**	Rho GEF	**Y**	SVIL	Actin binding protein	
FHDC1	Formin		WASF1	WAVE complex	
FHOD1	Formin		WASF2	WAVE complex	
FHOD3	Formin		WASF3	WAVE complex	
**FLNA**	Filament crosslinking		WASL	WAVE complex	
FMN1	Formin		WDR1	Filament disassembly	Y

Table S1. List of genes included in RNAi screen for mitotic rounding and main hits.

A list of the 60 genes screened for defects in mitotic shape, with hits indicated with orange shading. Proteins that have been suggested to be phosphorylated specifically at mitosis or to be substrates of mitotic kinases are indicated in bold text. Out of the major hits, Ect2 was the only mitotic kinase substrate that was identified as a regulator of mitotic shape. As well as Ect2, two other regulators of mitotic rounding were identified. WDR1, which has been previously shown to control actin in mitosis (Fujibuchi et al., 2005) and CAPZB, a component of the F-actin capping complex which binds the fast growing filament barbed ends to prevent monomer exchange, thus regulating filament length and dynamics (Pollard and Cooper, 2009).

SUPPLEMENTAL EXPERIMENTAL PROCEDURES

Cell culture and transfection

HeLa-Kyoto cells were cultured in Dulbecco's Modified Eagle Medium (DMEM, Gibco) with 10% fetal bovine serum and 1% Pen-Strep (Sigma-Aldrich). Lipofectamine 2000 (Invitrogen) was used for siRNA transfections according to the manufacturer's protocol. Cells were analyzed 24 hrs after transfection. siRNAs were used to knock down RacGAP1 (Dharmacon D-008650-01 CAAATTATCTCTGAAGTGT and D-008650-04 GTAATCAGGTGGATGTAGA), Mad2 (Dharmacon D-003271-15 GGTTGTAGTT ATCTCAAAT) and Ect2 (Qiagen Hs-Ect2-5 TTGCCTAGAGATAGCAAGAAA, Hs-Ect2-6 ATGACGCATATTAATGAGGAT, and Hs-Ect2-9 AAGAGTAAGGTTTACCTGTTA) and compared to a non-targeting control (Qiagen AllStars Negative Control siRNA). Most experiments were carried out in duplicate using Hs-Ect2-5 and Hs-Ect2-6. Images used in figures show Hs-Ect2-6, unless otherwise stated. For plasmid DNA transfection, Fugene HD (Roche) was used according to the manufacturer's instructions. The following constructs were used: Tubulin-mRFP(Kobayashi and Murayama, 2009), Ect2-FL-GFP (Niiya et al., 2006) and Ect2-C-GFP (Su et al., 2011) and Ect2-C-myc (Yuce et al., 2005). Ect2-dNLS-GFP was constructed by mutating the 2 NLS sites (RKRRRLK>RKAAALK and PRKRP>PAKAP) according to (Saito et al., 2004) using the QuikChange Site Directed Mutagenesis kit (Stratagene). Ect2V566D and Ect2T342D-S366D-GFP were constructed using the QuikChange Site Directed Mutagenesis kit (Stratagene), using full length Ect2-GFP as a template. To generate a stable HeLa cell expressing lifeAct-GFP (Riedl et al., 2008) and histone2B-mRFP (Keppler et al., 2006), plasmids were transfected and then after 48 hours, selection media containing 0.5 μg/ml G418 and 5 μg/ml puromycin was added and cells serially diluted. Individual colonies of stable double-positive clones were then isolated using cloning circles.

RNAi screen for regulators of mitotic rounding

To identify regulators of mitotic rounding, a targeted library of siRNAs was used to silence 60 genes. Given the role of the actin cytoskeleton in mitotic rounding (Kunda and Baum, 2009), we selected genes with known roles in actin regulation. This included core regulators of F-actin dynamics including filament nucleators as well as genes required for filament capping, severing and bundling. We also included a number of upstream regulators of actin, including the WAVE complex and Rho GTPases. In addition, a number of extra actin regulators were included because they had been identified in large-scale screens as containing Cdk1 phosphorylation sites or as being specifically phosphorylated at mitosis (Blethrow et al., 2008; Dephoure et al., 2008; Ji et al., 2002; Lowery et al., 2007; Xiang et al., 2008). Four different non-overlapping siRNA oligonucleotides (Dharmacon) were used for each gene. In addition, siRNAs for known homologues were pooled. siRNAs were arrayed in 96 well plates (PerkinElmer) by hand and then transferred in quaduplicate into 384 well plates using a liquid handling robot (Tecan). A reverse transfection was then carried out using Lipofectamine 2000 (Invitrogen) according to the manufacturer's protocol and 2,500 HeLa cells were added to each well. Cells were incubated with siRNAs for 72 hours before fixation. 8 hours before fixation 5 μM s-trityl L-cysteine (STLC, Sigma) was added to two of the four replicates of each siRNA. This caused cells to arrest in mitosis to ensure a large number of mitotic cells for analysis. However, phenotypes observed in STLC treated cells were verified in mitotic cells in the untreated wells to control against any defects in mitotic

shape induced by the drug. After 72 hours, cells were fixed with 4% paraformaldehyde, washed with PBS and permeablised with 0.2% triton-X/PBS. After a half hour blocking step in 5% Bovine Serum Albumin/PBS, cells were stained with TRITC-conjugated phalloidin (Sigma) to label actin, a FITC-conjugated anti-tubulin Antibody (DM1A, Sigma) and DAPI (Invitrogen) to identify mitotic cells. Plates were then imaged and automatically analysed using an Opera high content screening platform (Perkin Elmer). Plates were imaged using a 20x objective for automated analysis. Images were analysed using a script developed in the PerkinElmer Acapella software. Briefly, nuclei were segmented and mitotic nuclei were identified based on their size and brightness. Mitotic cell bodies were then segmented in the actin channel and the width/length ratio of cells measured. siRNAs were scored based on the mean width/length ratio compared to non-targeting control as a measure of the roundness of mitotic cells. In addition, cells were imaged using a 60x objective and every siRNA was inspected visually, following randomisation of wells to ensure a non-biased analysis, and scored for defects in cells shape and actin distribution. Hits were determined based on the sum of the width/length score and visual score for all for four siRNAs for each gene. Genes were only classed as hits if at least 2 siRNAs displayed the same phenotype. Hits were then validated by time lapse imaging of siRNA treated cells and western blotting to confirm protein knockdown where antibodies were available. A list of genes screened and hits is displayed in table S1.

SUPPLEMENTAL REFERENCES

Blethrow, J.D., Glavy, J.S., Morgan, D.O., and Shokat, K.M. (2008). Covalent capture of kinase-specific phosphopeptides reveals Cdk1-cyclin B substrates. Proceedings of the National Academy of Sciences of the United States of America *105*, 1442-1447.

Dephoure, N., Zhou, C., Villen, J., Beausoleil, S.A., Bakalarski, C.E., Elledge, S.J., and Gygi, S.P. (2008). A quantitative atlas of mitotic phosphorylation. Proceedings of the National Academy of Sciences of the United States of America *105*, 10762-10767.

Fujibuchi, T., Abe, Y., Takeuchi, T., Imai, Y., Kamei, Y., Murase, R., Ueda, N., Shigemoto, K., Yamamoto, H., and Kito, K. (2005). AIP1/WDR1 supports mitotic cell rounding. Biochemical and biophysical research communications *327*, 268-275.

Ji, J.Y., Haghnia, M., Trusty, C., Goldstein, L.S., and Schubiger, G. (2002). A genetic screen for suppressors and enhancers of the Drosophila cdk1-cyclin B identifies maternal factors that regulate microtubule and microfilament stability. Genetics *162*, 1179-1195.

Keppler, A., Arrivoli, C., Sironi, L., and Ellenberg, J. (2006). Fluorophores for live cell imaging of AGT fusion proteins across the visible spectrum. BioTechniques *41*, 167-170, 172, 174-165.

Lowery, D.M., Clauser, K.R., Hjerrild, M., Lim, D., Alexander, J., Kishi, K., Ong, S.E., Gammeltoft, S., Carr, S.A., and Yaffe, M.B. (2007). Proteomic screen defines the Polo-box domain interactome and identifies Rock2 as a Plk1 substrate. The EMBO journal *26*, 2262-2273.

Pollard, T.D., and Cooper, J.A. (2009). Actin, a central player in cell shape and movement. Science (New York, NY *326*, 1208-1212.

Xiang, M., Xue, C., Huicai, L., Jin, L., Hong, L., and Dacheng, H. (2008). Large-scale identification of novel mitosis-specific phosphoproteins. Biochimica et biophysica acta *1784*, 882-890.
