# Supplementary figures and images for: Changes in Ect2 Localization Couple Actomyosin-Dependent Cell Shape Changes to Mitotic Progression
Source: Dev Cell. 2012 Aug 14;23(2):371–83. doi: 10.1016/j.devcel.2012.06.003 (PMC3763371; doi:10.1016/j.devcel.2012.06.003)

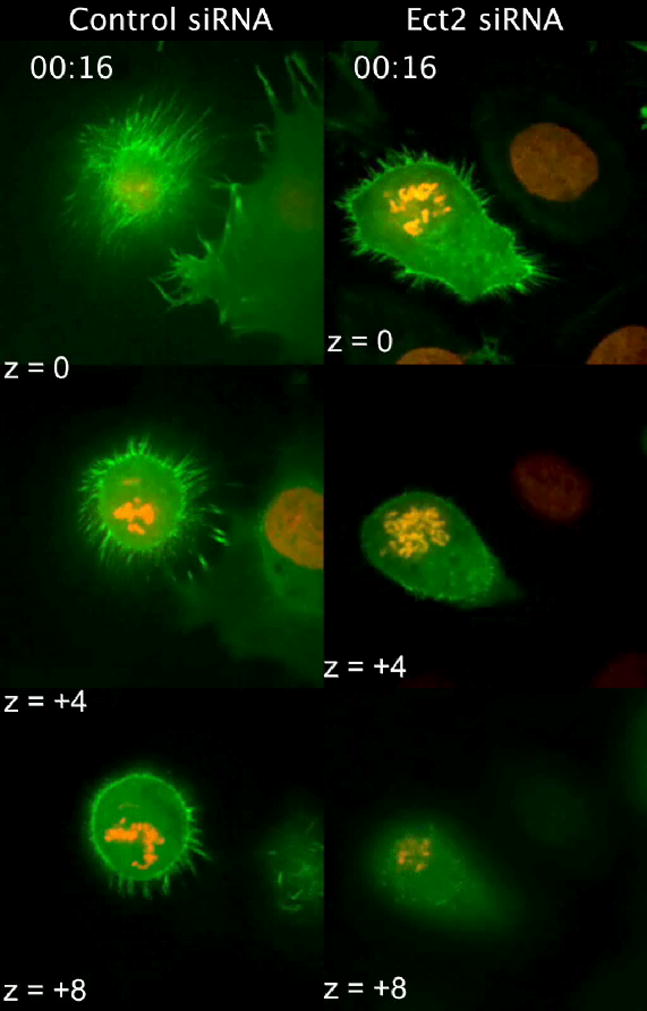

Supplement: Movie S1. HeLa Cells Treated with Control siRNA and Ect2 siRNA and Labelled with LiveAct-eGFP and Histone H2B-mcherry Round Up as They Enter Mitosis, Related to Figures 2A and 2B [file mmc2.jpg]

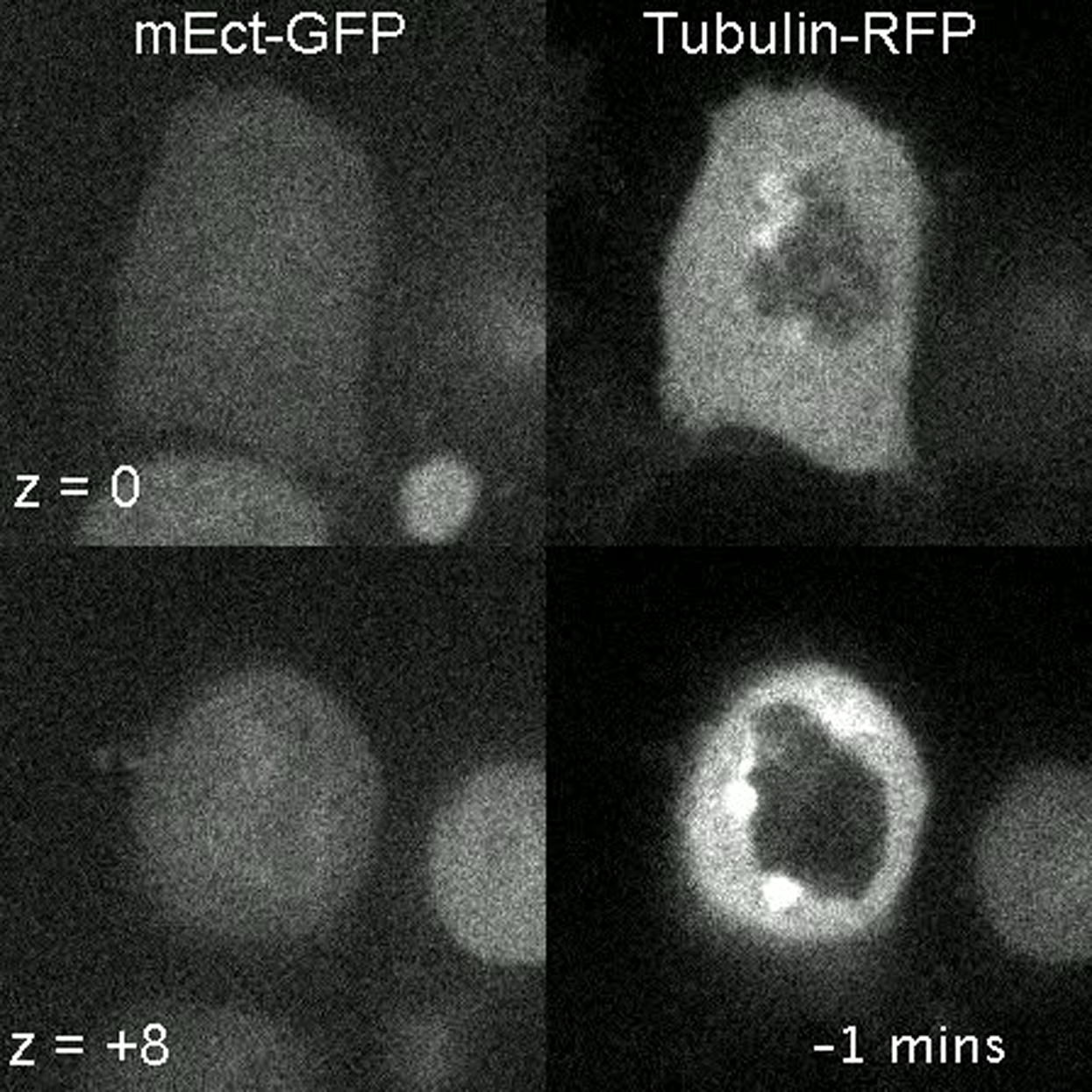

Supplement: Movie S2. HeLa Cell Expressing Mouse-Ect2-GFP and Tubulin-mRFP Rounds Up and Enters Mitosis, Related to Figures 5B and 5C [file mmc3.jpg]

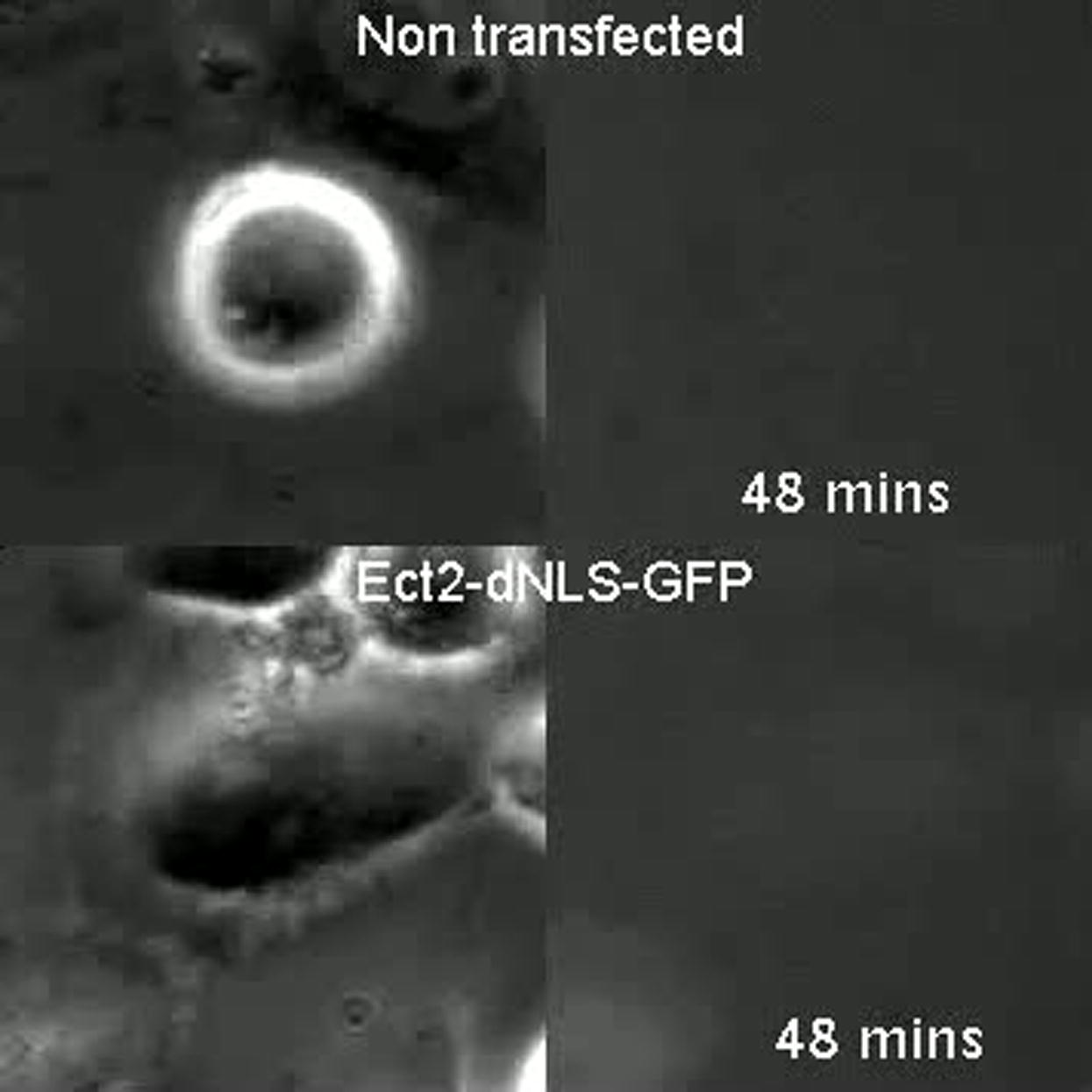

Supplement: Movie S3. Phase Contrast and GFP Fluorescence Movies of a Nontransfected HeLa Cell and a Cell Transfected with Ect2-dNLS-GFP Rounding Up as They Progress through Mitosis, Related to Figure 6F [file mmc4.jpg]
